# A new species of *Peritresius* Leidy, 1856 (Testudines: Pan-Cheloniidae) from the Late Cretaceous (Campanian) of Alabama, USA, and the occurrence of the genus within the Mississippi Embayment of North America

**DOI:** 10.1371/journal.pone.0195651

**Published:** 2018-04-18

**Authors:** Andrew D. Gentry, James F. Parham, Dana J. Ehret, Jun A. Ebersole

**Affiliations:** 1 Department of Biology, University of Alabama at Birmingham, Birmingham, Alabama, United States of America; 2 Department of Geological Sciences, California State University Fullerton, Fullerton, California, United States of America; 3 New Jersey State Museum, Trenton, New Jersey, United States of America; 4 Department of Collections, McWane Science Center, Birmingham, Alabama, United States of America; Royal Belgian Institute of Natural Sciences, BELGIUM

## Abstract

Late Cretaceous members of *Peritresius* belong to a diverse clade of marine adapted turtles currently thought to be some of the earliest representatives of the lineage leading to modern hard-shelled sea turtles (Pan-Cheloniidae). Prior studies have suggested that *Peritresius* was monospecific, with a distribution restricted to Maastrichtian deposits in North America. However, new *Peritresius* specimens identified from Alabama and Mississippi, USA, show that the genus contains two taxa, *Peritresius ornatus*, and a new species *Peritresius martini* sp. nov. These two taxa are characterized by the presence of a generally cordiform carapace with moderately serrated peripherals, well-developed ventral flanges beginning at the third peripheral, squarish umbilical and lateral plastral fontanelles, and a narrow bridge formed by the contact between the hyoplastron and hypoplastron. *Peritresius martini* sp. nov. can be distinguished by its lack of dermal ornamentation and the presence of a ‘rib-free’ 10th peripheral. These new specimens represent the first occurrences of *Peritresius* from the Late Cretaceous Mississippi Embayment and extend the temporal range of this genus back to the early Campanian. When tested within a global phylogenetic context, *Peritresius* is placed on the stem of Cheloniidae (Pan-Cheloniidae) along with *Ctenochelys* and *Allopleuron hofmanni*. The heavily vascularized and uniquely sculptured dermal elements of *P*. *ornatus* are interpreted here as potentially relating to thermoregulation and therefore may have been one of the key factors contributing to the survival of *Peritresius* into the Maastrichtian, a period of cooling when other lineages of Campanian marine turtles (e.g., Protostegids, *Toxochelys*, and *Ctenochelys*) went extinct.

## Introduction

Cretaceous marine turtle fossils are abundant within Santonian to Campanian marine deposits in the southeastern United States, and have been reported from Alabama, Arkansas, Georgia, Mississippi, and Tennessee [1–4]. Although extensive Maastrichtian surface deposits are present in these southern states, few marine turtle specimens have been recovered from these units [5]. To date, the only well-described marine turtle known definitively from the Maastrichtian of the southeastern United States is *Peritresius ornatus* Baird, 1964 [4], a taxon reported previously from only the Navesink and Redbank Formations of New Jersey and the Ripley Formation of Georgia ([4,6]). The most frequently recovered Cretaceous marine turtle taxa from the Cretaceous of the southeastern U.S. are *Toxochelys* Cope, 1873 [7], *Ctenochelys* Zangerl, 1953 [2] and *Prionochelys* Zangerl 1953 [2], with each genus represented by dozens, or in the case of *Toxochelys*, hundreds of specimens. Despite their abundance in the southeastern U.S., these genera appear absent from the Maastrichtian components of the Hornerstown and Navesink formations along the Atlantic Coast, and are seemingly absent entirely from Maastrichtian deposits in North America (Zangerl 1953 [2]). The relative paucity of Maastrichtian pan-chelonioids (i.e., *P*. *ornatus*, *Catapleura* Cope, 1868 [8], *Euclastes* Cope, 1867 [9]) and their relationship to well-known Santonian and Campanian taxa (i.e. *Toxochelys latiremis* Cope, 1873 [7] and *Ctenochelys stenoporus* Zangerl, 1953 [2]), are both issues of particular interest to any attempt to resolve the phylogeny and biogeography of Late Cretaceous Pan-Chelonioidea.

Recently, the remains of several marine turtles referred to the genus *Peritresius* Leidy, 1856 [10], including those of a new species, were identified in the collections at the Alabama Museum of Natural History in Tuscaloosa, USA, McWane Science Center in Birmingham, Alabama, USA, and the Mississippi Museum of Natural Sciences in Jackson, USA. Presented herein are descriptions of these specimens along with comments on their taxonomy. We also provide remarks on chelonioid diversity and paleobiogeography during the Late Cretaceous.

## Geologic setting

**Fig 1:**
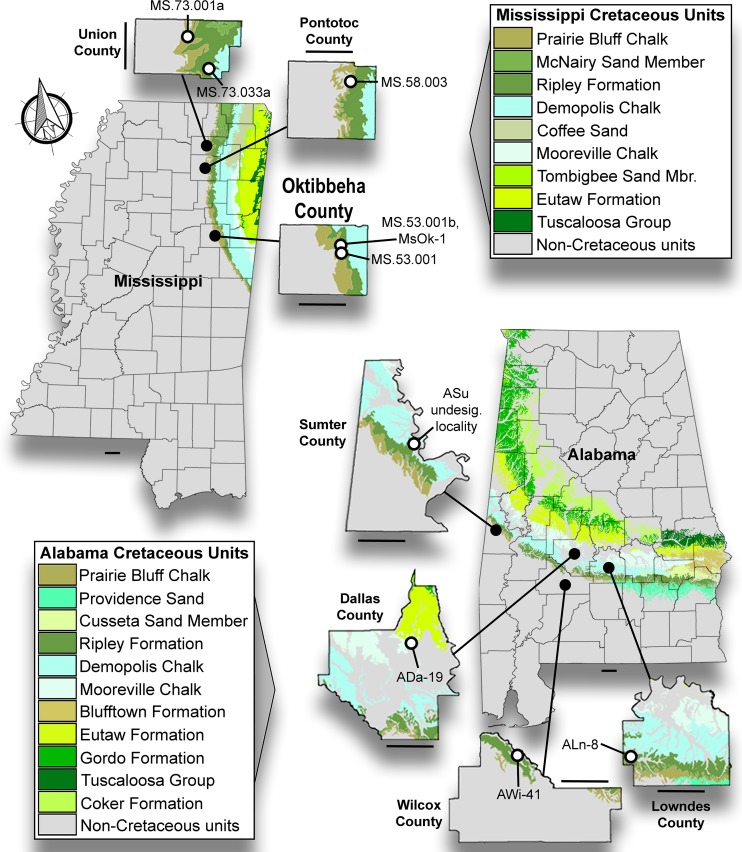
Surface stratigraphy of Alabama and Mississippi *Peritresius* localities. Upper Cretaceous surface exposures in both Alabama and Mississippi and the localization of discussed *Peritresius* specimens.

**Fig 2:**
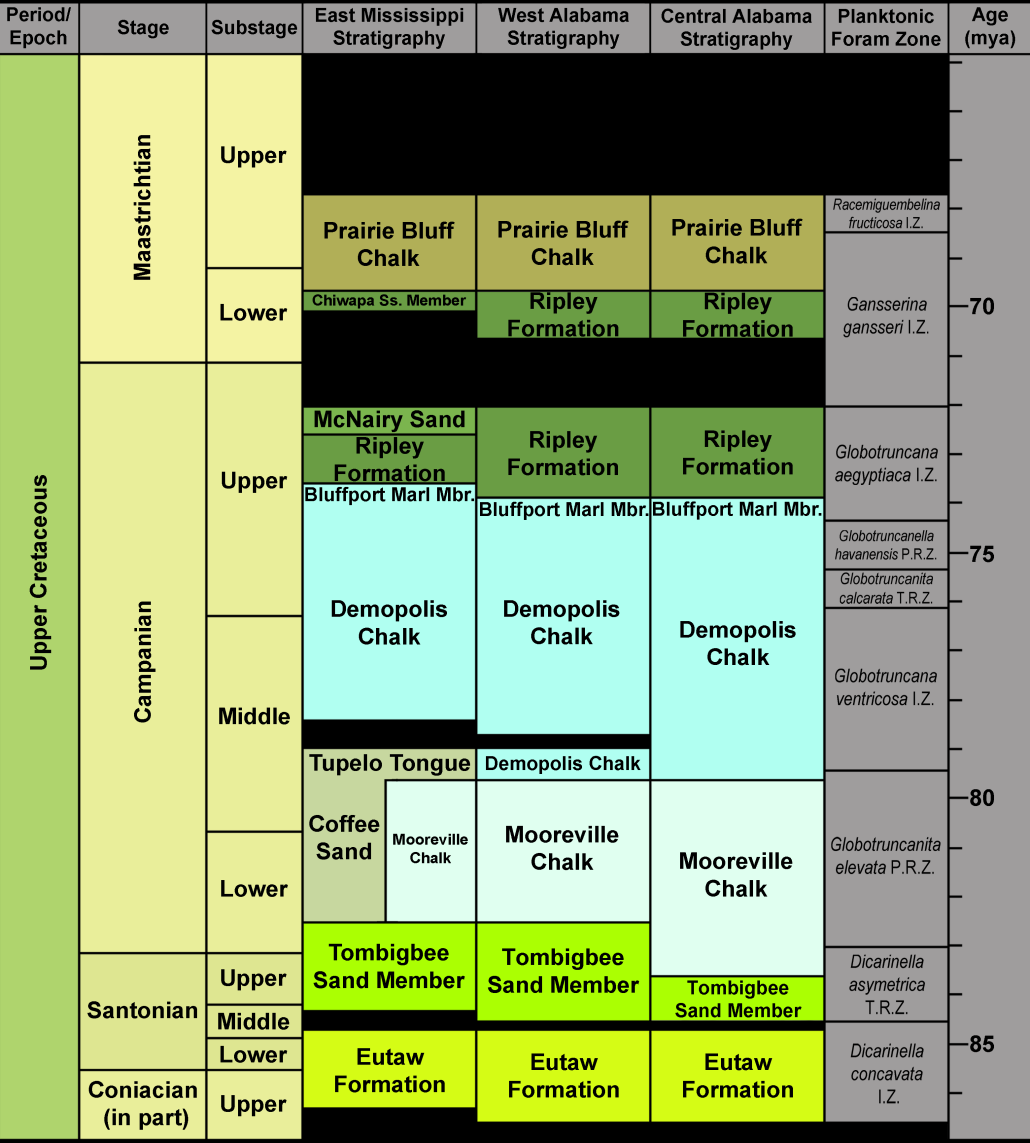
Generalized Santonian through Maastrichtian surface stratigraphy in west and central Alabama and east Mississippi. Stratigraphy follows that of Mancini *et al*. [11] and Dockery [12]. Planktonic foraminiferal zones after Caron [13].

The specimens examined in this study were collected from 10 distinct localities spread across seven counties in eastern Mississippi and central and western Alabama, USA (Fig 1). The specimens were derived from four Upper Cretaceous formations, the Mooreville Chalk, Demopolis Chalk, Ripley Formation, and Prairie Bluff Chalk, which span from the lower Campanian through the upper Maastrichtian (Fig 2).

Although the lithologies of these formations vary considerably, their depositional settings are similar as all are interpreted to represent outer neritic to nearshore environments ([14–15]; see Table 1).

**Table 1:** List of relevant stratigraphic units, localities, and specimens. (AL) Alabama. (MS) Mississippi. Lithologic descriptions and depositional environments follow Raymond et al. [14] and Puckett [15].

Age	Unit	Lithology	Depositional Setting	Locality	County	State	Specimens
Maastrichtian	Prairie Bluff Chalk	Firm sandy, fossiliferous, brittle chalk	Middle to outer neritic environments	MS.53.001b	Oktibbeha	MS	*P*. *ornatus*: MMNS 5533, MMNS 5710
				MS.58.003	Ponototoc	MS	*P*. *ornatus*: MMNS 4546
				MS.53.001	Oktibbeha	MS	*P*. *ornatus*: MMNS 4003, MMNS 4547
				Unknown	Sumter	AL	*P*. *ornatus*: ALMNH 5497
Campanian	Ripley Formation	Thin indurated beds of fossiliferous sandstone, sandy calcareous clay, and massive, micaceous, glauconitic, fossiliferous fine sand	Majority deposited under lower shore-face conditions	**ALn-8, Type Locality**	Lowndes	AL	***P*. *martini* sp. nov.: ALMNH 6191**
							*P*. *ornatus*: ALMNH 8988
				AWi-41	Wilcox	AL	*P*. *ornatus*: MSC 5741
				MS.73.033	Union	MS	*P*. *ornatus*: MMNS 5102, MMNS 5274, MMNS 7521, MMNS 8632.4
				MS.73.001a	Union	MS	*P*. *ornatus*: MMNS 5876
	Bluffport Marl Member of the Demopolis Chalk	Compact brittle chalk overlain by abundantly fossiliferous chalky marl, clayey chalk, and calcareous clay	Middle neritic environments	ASu- undesignated locality	Sumter	AL	*P*. *ornatus*: ALMNH 5887
	Demopolis Chalk	Clayey, very finely sandy, micaceous chalk	Middle to outer neritic environments	MsOk-1	Oktibbeha	MS	*P*. *ornatus*: ALMNH 6256
				Unknown	Dallas	AL	*P*. *ornatus*: ALMNH 3900
	Mooreville Chalk	Compact fossiliferous clayey chalk and chalky marl	Inner to middle neritic environments below the wave base	ADa-19	Dallas	AL	*P*. *ornatus*: ALMNH 3780

The holotype specimen described herein (ALMNH 6191) was surface collected from Ripley Formation exposures at site ALn-8, a creek locality located in Lowndes County, Alabama (Fig 1). In Alabama, an unconformity exists within the Ripley Formation, dividing it into upper and lower components (see Fig 2). The vertebrate remains from site ALn-8 fall were collected from the *Gansserina gansseri* (Bolli, 1951 [16]) Planktonic Foraminiferal Interval Zone, indicating they were recovered from the lower Ripley Formation, making them upper Campanian in age. The surface geology at site ALn-8 was described by Hall and Savrda [17], and this site is known for producing large numbers of fossil-bearing phosphatic concretions that contain crabs, spiny lobsters, and occasionally vertebrate remains. At the same time, however, vertebrate remains not encased in concretions occur only intermittently within the exposures.

Across Alabama, numerous Late Cretaceous vertebrate taxa have been reported from the upper Campanian to lower Maastrichtian Ripley Formation including sharks (i.e. *Brachyrhizodus* sp., *Cretalamna* sp., *Ginglymostoma* sp., *Pseudocorax laevis* (Leriche, 1906 [18]), *Scapanorhynchus texanus* (Roemer, 1849 [19]), and *Squalicorax pristodontus* (Agassiz, 1843 [20]), bony fishes (i.e. *Anomoeodus* sp., *Xiphactinus audax* Leidy, 1870 [21], *Enchodus ferox* Leidy, 1855 [22], and *Enchodus petrosus* Cope 1874 [23], a crocodile (i.e. *Deinosuchus rugosus* Emmons, 1858 [24]), mosasaurs (i.e. *Mosasaurus maximus* Cope, 1869 [25] and *Plioplatecarpus* sp.), and marine turtles (i.e. *Ctenochelys* sp. and *Protostega gigas* Cope 1871 [26]) [5,27]. As part of this study, two additional marine turtles have been identified within this formation, *Peritresius ornatus* (Baird, 1964 [4]) and a new taxon described herein, *Peritresius martini* sp. nov. Although this new taxon is currently known only from the type locality, here we report the occurrence of *P*. *ornatus* from nine additional late Campanian to lower Maastrichtian localities within Alabama and Mississippi. Information regarding the strata exposed at these localities is listed in Table 1.

Within this study, all localities are referenced by standard Alabama and Mississippi site file numbers. All cited localities are located on private property; however, permission was obtained by the ALMNH, MMNS, and MSC to collect at these locations. All specimens are legal property of the specific museums. Precise locality information is not provided herein though this information is fully available to qualified researchers and is on file at the ALMNH, MMNS, and MSC.

### Nomenclatural acts

The electronic edition of this article conforms to the requirements of the amended International Code of Zoological Nomenclature, and hence the new names contained herein are available under that Code from the electronic edition of this article. This published work and the nomenclatural acts it contains have been registered in ZooBank, the online registration system for the ICZN. The ZooBank LSIDs (Life Science Identifiers) can be resolved and the associated information viewed through any standard web browser by appending the LSID to the prefix "http://zoobank.org/". The LSID for this publication is: urn:lsid:zoobank.org:pub:815E2FD7-146C-4E13-896E-080BC9B683B2. The electronic edition of this work was published in a journal with an ISSN, and has been archived and is available from the following digital repositories: PubMed Central and LOCKSS.

## Materials and methods

### Cladistic methods and taxonomy

The character-taxon matrix used in this study (S1 File) follows that of Cadena and Parham [28], and was modified to include scores for *Peritresius* and recently discussed character adjustments for *Ctenochelys* and *Toxochelys latiremis* ([29], see S2 File). The character state of ‘absent’ was removed from character 133 (rib-free peripherals) to reflect the original states proposed for this feature (see [30], ch. 21). The 37 ordered characters used in Cadena and Parham [28] were also used in the present study. Terminal operational taxonomic units (OTUs) were limited to individual species. Cretaceous fossil taxa were restricted to only those that could be adequately incorporated into the matrix (more than 30% of characters coded). The only excluded Cretaceous marine turtle meeting this minimum threshold was *Euclastes wielandi* (Hay, 1908 [31]). This was due to the fact that the inclusion of *Euclastes wielandi* caused all crown cheloniids to collapse into a polytomy. The fact that *Euclastes wielandi* is known exclusively from cranial material may contribute to its behavior as a rogue taxon in our matrix. The matrix was analyzed with PAUP* v4.0 with all characters considered equally weighted using the heuristic search function and the subtree pruning/regrafting method of rearrangement. Bootstrap values were based on 1000 replicates and decay indices were calculated by retaining trees with sequentially higher steps than the most parsimonious strict-consensus tree until all bipartitions had collapsed. The positions of extant taxa were constrained by an incorporated ‘molecular scaffold’ (S3 File) taken from global phylogenomic studies of turtles [32]. Phylogenetic nomenclature and definitions follow Joyce [33] and Joyce et al. [34]. Osteological terminology largely follows that of Gaffney [35], but includes recent adjustments to the terminology for the carotid arteries [36]. Numbers in parentheses refer to characters used in the phylogenetic analyses and their corresponding scores.

## Systematic Paleontology

**Reptilia** Laurenti, 1768 [37]

**Testudines** Batsch, 1788 [38]

**Cryptodira** Cope, 1868 [8]

**Chelonioidea** Baur, 1893 [39]

**Pan-Cheloniidae** Joyce, Parham, and Gauthier, 2004 [40]

Genus ***Peritresius*** Leidy, 1856 [10]

### Type species

*Peritresius ornatus* Baird, 1964 [4], figs 1–8, Navesink Formation (upper Maastrichtian), Burlington County, New Jersey, USA.

### Amended diagnosis

Cretaceous pan-cheloniid differentiated from *Allopleuron hofmanni* in having a more broadly rounded carapace, a decreased distance between the axillary and inguinal notches of the plastron, a lack of elongate, finger-like lateral projections of the hypoplastron, and the relatively constant width of peripheral elements 3–11. Differentiated from pan-cheloniids such as *Ctenochelys* by a greatly expanded contact between the left and right epiplastra, significantly reduced contact between the hyo- and hypoplastron due to the presence of large central and lateral plastral fontanelles, and a highly domed carapace as evidenced by the broad angle (90°-120°) formed by the dorsal and ventral facets of peripherals 3–8. Specimens can be diagnosed as *Peritresius* by the following combination of features: generally cordiform carapace having peripheral elements with moderate lateral serrations; a single mid-sagittal keel on the dorsal surface of the carapace (ch.116/3) consisting of 7 keeled neurals (ch.126/1) with epineural ossifications situated at the junctures of neurals 3–4, 5–6, and 7-suprapygal 1; reduction in peripheral height moving posteriorly from peripheral 4; ratio between the axillary-inguinal distance of the plastron and the length of hyo-hypoplastral contact >2.5:1 (plastral index is this value * 100); and thyroid fenestra subdivided by pronounced contact between the pubes and ischia (ch.224/1).

***Peritresius martini*** sp. nov.

urn:lsid:zoobank.org:act:B876072A-57AE-4603-AAFC-D169B325E204

Figs 3–5

**Fig 3:**
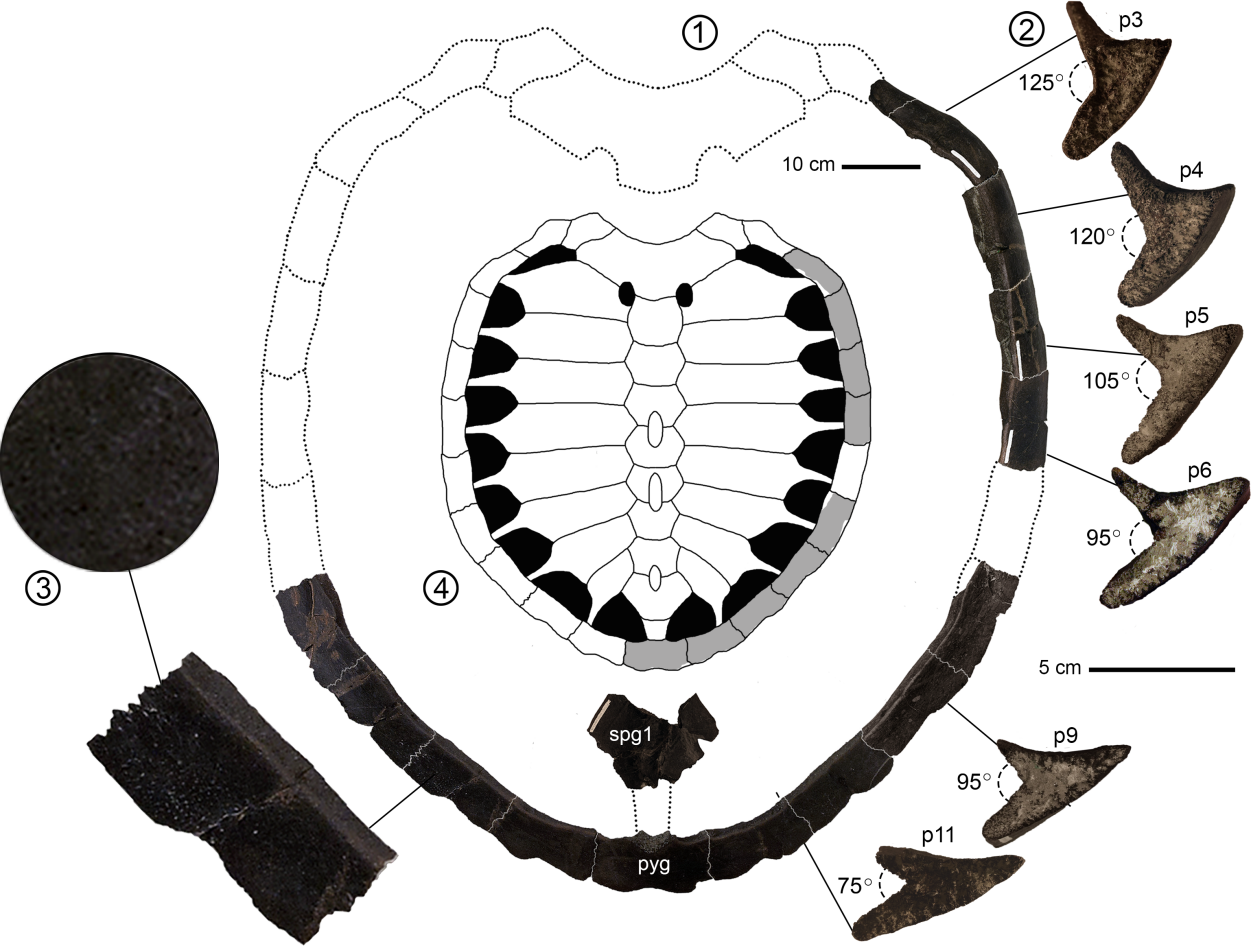
*Peritresius martini* sp. nov., carapace, ALMNH 6191 (holotype) from the upper Campanian of Alabama, USA. (1) carapace in dorsal view and plastron in ventral view; (2) left peripherals 3–6, 9, & 11 in posterior view; (3) 10X magnified view of the dorsal surface of right peripheral 10; (4) hypothetical reconstruction of the complete shell with the preserved elements shown in gray. Abbreviations: p, peripheral; pyg, pygal; spg, suprapygal.

**Fig 4:**
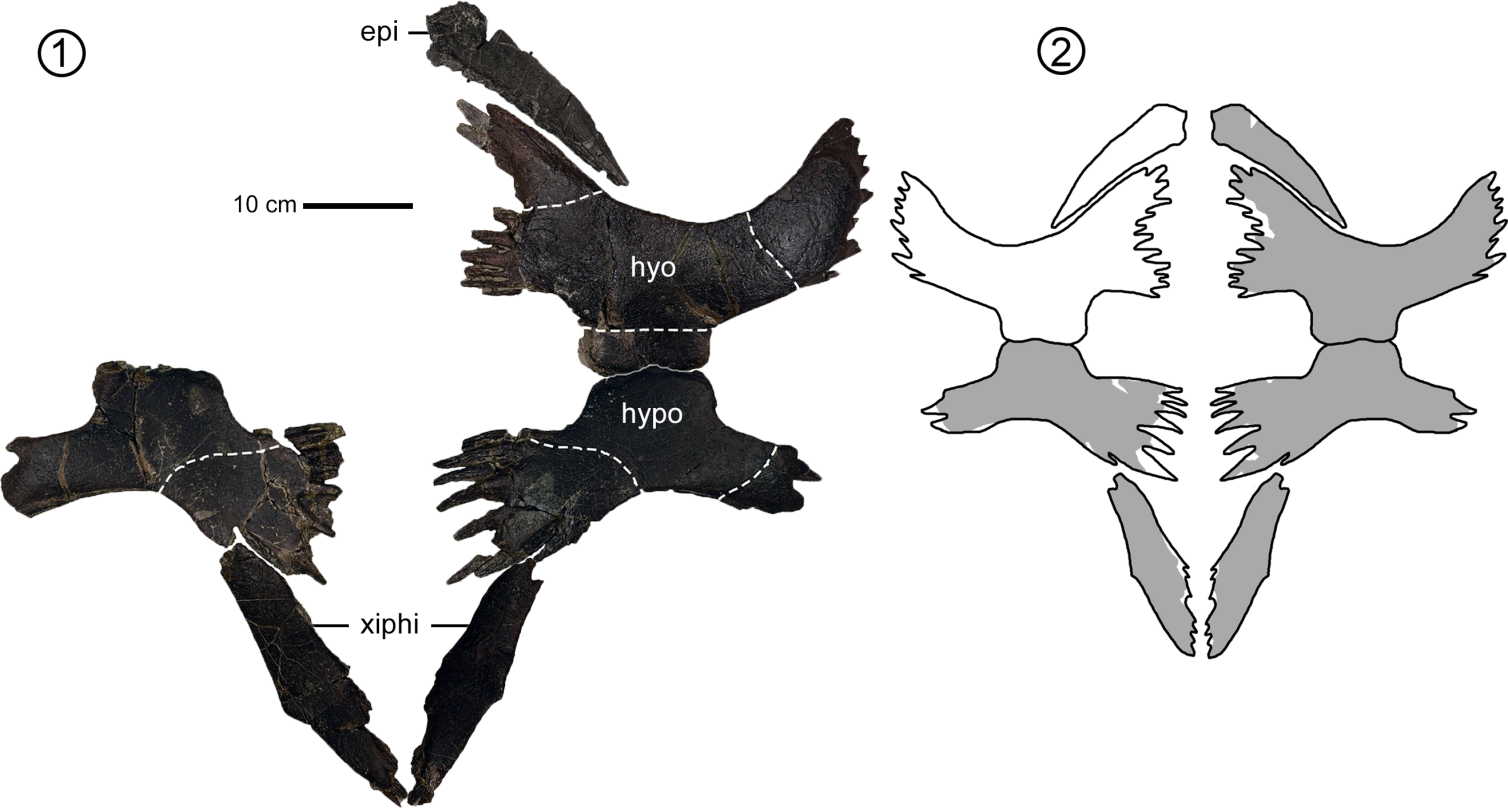
*Peritresius martini* sp. nov., plastron, ALMNH 6191 (holotype) from the upper Campanian of Alabama, USA. (1) Plastron in ventral view; (2) hypothetical reconstruction of the plastron with the preserved elements shown in gray. Abbreviations: epi, epiplastron; hyo, hyoplastron; hypo, hypoplastron; xiphi, xiphiplastron.

**Fig 5:**
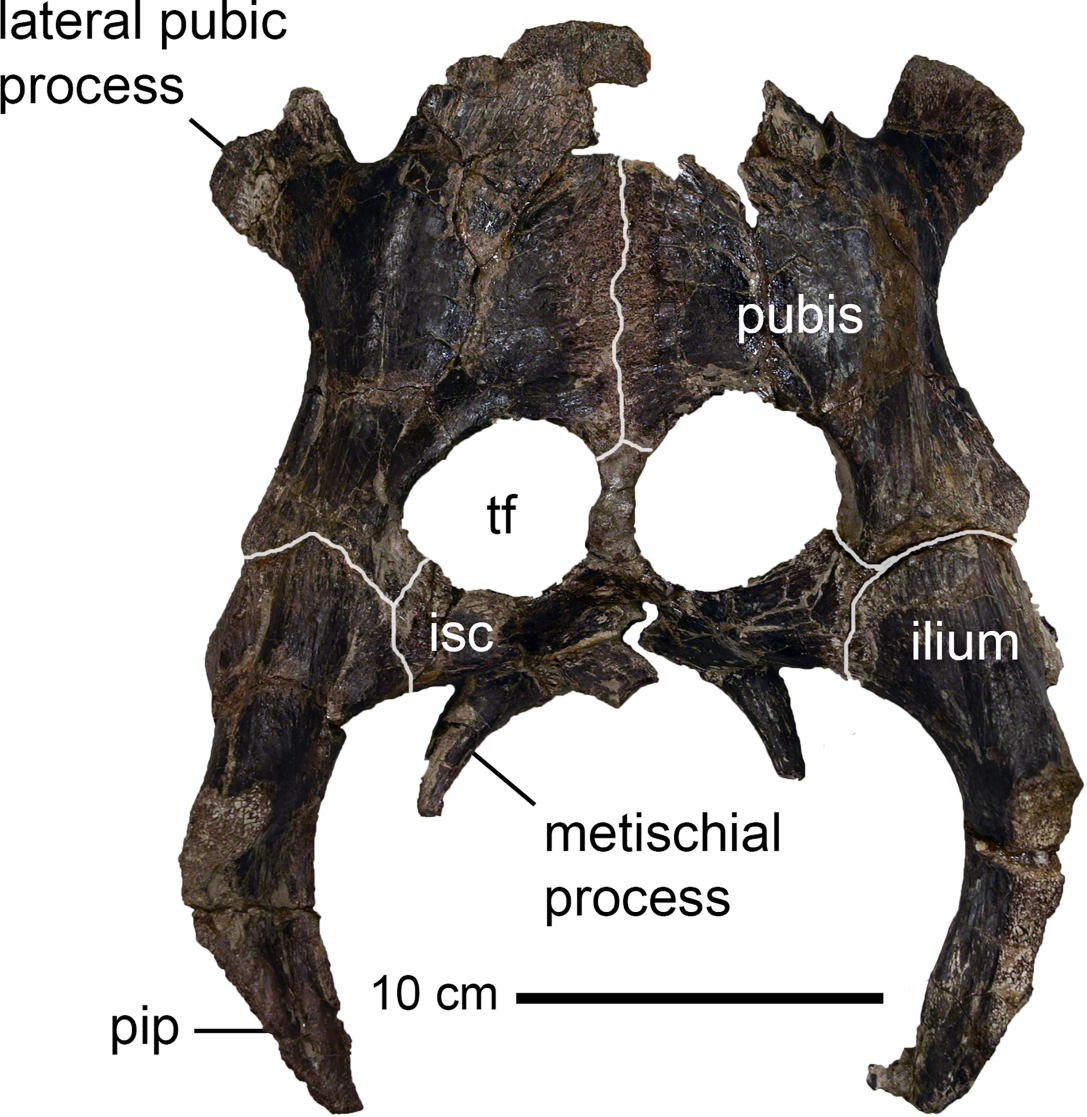
*Peritresius martini* sp. nov., pelvis, ALMNH 6191 (holotype) from the upper Campanian of Alabama, USA. Pelvis in dorsal view. Abbreviations: isc, ischium; pip, posterior iliac process; tf, thyroid fenestra.

### Etymology

*martini*: for the discoverer and initial preparator of the holotype specimen, Mr. George Martin of Auburn, Alabama.

### Differential diagnosis

As for genus but can be distinguished from *Peritresius ornatus* by a lack of sculpturing on the dermal surfaces of the carapacial elements, a less pronounced lateral keel of the anterior peripherals, and a ‘rib-free’ 10^th^ peripheral (ch.133/2).

### Holotype

ALMNH 6191 (Figs 3–5), includes peripherals 3–6 and 8–11 of the right side, peripherals 8–11 of the left side, pygal, partial 1^st^ suprapygal, right epiplastron, right hyoplastron, both hypoplastra, both xiphiplastra, and an articulated pelvic girdle.

### Type locality

Site ALn-8, Dry Cedar Creek, Lowndes County, Alabama, USA.

### Type stratum

Lower Ripley Formation, lower *Globotruncana aegyptiaca* Interval Zone, upper Campanian.

## Description of *P*. *martini* sp. nov.

### Carapace

The preserved region of the carapace allows us to interpret a morphology typical for Cretaceous pan-cheloniids in having a broadly cordiform general outline and moderately serrated peripherals (Fig 3). ALMNH 6191 has an estimated total carapace length in excess of 90 cm, far exceeding the largest described specimen of *Peritresius ornatus* (NJSM 11051) the only other member of the genus. The greatest width of the carapace is interpreted as approximately 75 cm along a line between the posterior margins of the sixth peripherals. Prominent scale sulci can be seen on the dorsal face of the peripherals of ALMNH 6191 and appear to closely resemble the arrangement seen in *P*. *ornatus* (Fig 3; [4], figs 3–4). Unfortunately, an accurate rendering of the scutes across the entirety of the carapace is not possible given the partial nature of specimen. The dorsal surface of the carapace of *P*. *martini* lacks the vermiculate arrangement of papillae and rugosities characteristic of *P*. *ornatus*, however there appears to be some evidence of vascular innervations in the outermost cortical lamellae of the carapacial elements similar to that seen on *Corsochelys haliniches* Zangerl, 1960 [3] and certain Tertiary pan-cheloniids such as *Carolinochelys wilsoni* Hay, 1923 [41], (ChM PV4792, [42], fig 5).

### Peripherals

Although a complete peripheral series is not preserved with the holotype, enough of the peripherals are preserved that this portion of the carapace can be reconstructed with a reasonable degree of confidence. The peripheral series is typical for the genus (Fig 3) with each element marked by an interscutal sulcus on its dorsal surface and has a moderately serrated lateral margin similar to that of *Ctenochelys stenoporus* (Hay 1905 [43]) ([2], p. 240, fig 108). The lateral serrations begin with a large, circular boss on the first peripheral and they become more distinct and acute on the subsequent peripherals. Unlike *P*. *ornatus*, the lateral keel of *P*. *martini* is much less pronounced on the anterior peripherals. Along the medial and posterior peripherals, the keel crests anterior to the interscutal sulcus, similar to *Ctenochelys*. When viewed in anterior or posterior profile, the dorsal and ventral facets of peripherals 3–11 create a medially oriented trough that increases in height moving posteriorly from peripherals 3–5. The height increases until the fifth peripheral only to diminish posteriorly and terminate at the pygal (Fig 3). This arrangement is similar to that of *Allopleuron hofmanni* Gray 1831 [44] ([45], fig pl. 1, p. 41), however, unlike *A*. *hofmanni*, in dorsal view the peripherals of *P*. *martini* do not decrease in width moving posteriorly along the peripheral series. Beginning at the third peripheral, the medial trough is marked by shallow indentations that serve as insertion points for the distal ends of the adjacent costals.

As with *P*. *ornatus*, costal ribs 1–7 articulate with peripherals 3–9 but costal rib 8 articulates with peripheral 11 instead of 10. There is no indication of a rib-end insertion into the medial facet of the 10^th^ peripheral implying the presence of a rib-free peripheral between the 7^th^ and 8^th^ costal rib (ch. 133/2). This feature has been previously noted as a synapomorphy of the clade containing pan-cheloniids such as *Puppigerus* Cope, 1871 [26] and extant cheloniids [29,46]. The pygal is slightly notched at its posterior midpoint and transversely arched (Fig 3). The anterior margin of the pygal is marked by a large, circular articulation site for the posterior end of the 2^nd^ suprapygal.

### Plastral elements

The plastron of ALMNH 6191 is more complete than any previously described *Peritresius* specimen, lacking only the entoplastron, right hypoplastra and left epiplastra (Fig 4). There is no evidence of the dermal sculpturing observed on *P*. *ornatus*, though the narrow hyo-hypoplastral suture, large central and lateral plastral fontanelles, and orientation of the scute sulci make the plastron of *P*. *martini* more similar to that of *P*. *ornatus* than to any of the other closely related pan-cheloniids (i.e. *C*. *stenoporus—*[47], USNM 357166, fig 13C; [2], USNM 6013, fig 108; *Allopleuron hofmanni—*[45], pl. 33). However, it should be noted that the most intact plastra yet described of *C*. *stenoporus* ([47], USNM 357166; [2], USNM 6013) both belong to sub-adult individuals. Furthermore, greater than 50% of the *P*. *ornatus* plastron described by Baird ([4], NJSM 11051, figs 7–8) is plaster reconstruction.

The finely pointed interdigitations marking the lateral margins of the hyo- and hypoplastron are larger than those found on *C*. *stenoporus*, but are smaller than those of *A*. *hofmanni*. These interdigitations are indicative of a fully ligamentous connection between the carapace and plastron (ch. 148/1) as seen on juvenile *Lepidochelys* specimens (see [48], fig 83). The lateral and central plastral fontanelles are more expansive than those of other comparably sized Cretaceous pan-chelonioids (i.e. *Toxochelys* and *Ctenochelys*), a result of a diminished contact between the hyo- and hypoplastron (ch.153/1). Rather than flaring broadly both anteriorly and posteriorly (as in *Toxochelys* and *Ctenochelys*), the inguinal buttresses of the hypoplastron of *P*. *martini* are narrow and lie at nearly right angles to the midline, similar to that seen on *P*. *ornatus*. The orientation of the axillary and inguinal buttresses, along with the enlarged lateral fontanelles, create a bifid plastral connection with the carapace. The epiplastra are narrow and elongate, similar to those of *Ctenochelys*, and appear to have been suturally connected at their medial contact (ch. 160/1). The xiphiplastra are elongate and lack any significant medial sutural contact (ch. 169/2). The distal margins of the xiphiplastra are not as medially curved as in *Toxochelys* and *Ctenochelys*, but are instead quite straight and give the xiphiplastra an almost triangular appearance in ventral view, similar to the xiphiplastra of *A*. *hofmanni* ([45], pl. 32)

### Pelvis

Preserved with ALMNH 6191 is a nearly intact pelvic girdle (Fig 5). The arrangement of the girdle elements is typical of Cretaceous pan-cheloniids, such as *Ctenochelys*, and has large pubes and proportionally diminutive ischia. The thyroid fenestra is subdivided by an osseous contact between the posterior edge of the medial pubic processes and the anterior-most margin of the ischia (ch. 224/1), as seen on *A*. *hofmanni* ([45], pl. 43).

The medial processes of the ischia of ALMNH 6191 are less developed than those of *Toxochelys* but more so than those of Cenozoic cheloniids (ch. 233/1). The flattened lateral processes of the pubes are well developed (ch. 230/1) and slightly angled dorsally away from the medial plane of the symphyseal portion of the pubes, more so than observed in either *Toxochelys* or *Ctenochelys*. The posterior processes of the ilia are elongate and medially rugose, as on *Ctenochelys*, but also possess a dorsolateral rugosity potentially homologous with the ilio-carapacial contact observed in modern cheloniids. The remaining portions of the pelvis are indistinguishable from those of *Ctenochelys*.

### Remarks

A confluent thyroid fenestra has been suggested as a derived characteristic of crown cheloniids based on the subdivided thyroid fenestra of many early cryptodires and the absence of such a division in fossil chelonioids like *Toxochelys latiremis* and *Lophochelys* spp. Zangerl 1953 [2] ([49]). However, the presence of a divided thyroid fenestra in *Peritresius* spp., *A*. *hofmanni*, and certain extant cheloniids such as *Caretta caretta* ([48], figs 106a and 106b) may indicate this feature was lost early in pan-chelonioid evolution and later reacquired in select lineages of pan-cheloniids. It is also possible that Late Cretaceous sea turtles, such as *Toxochelys* and *Peritresius*, represent distinct radiations of marine adapted turtle potentially due to multiple invasions of marine environments by Testudines during the latter half of the Cretaceous, with the plesiomorphic condition retained in one lineage (*Peritresius*) and lost in another (*Toxochelys*). Testing the latter scenario using only morphology based phylogenetics would require an extensive review of the pelvic elements of fossil and extant Testudines in order to ensure that any character set or coding strategy regarding the arrangement of these elements was sufficiently inclusive to provide meaningful resolution between members of clades containing highly convergent lineages (i.e. marine turtles). Such a review is beyond the scope of the present study but is certainly an area of chelonioid evolution in need of further examination.

***Peritresius ornatus*** Leidy, 1856 [10]

(Figs 6–8)

**Fig 6:**
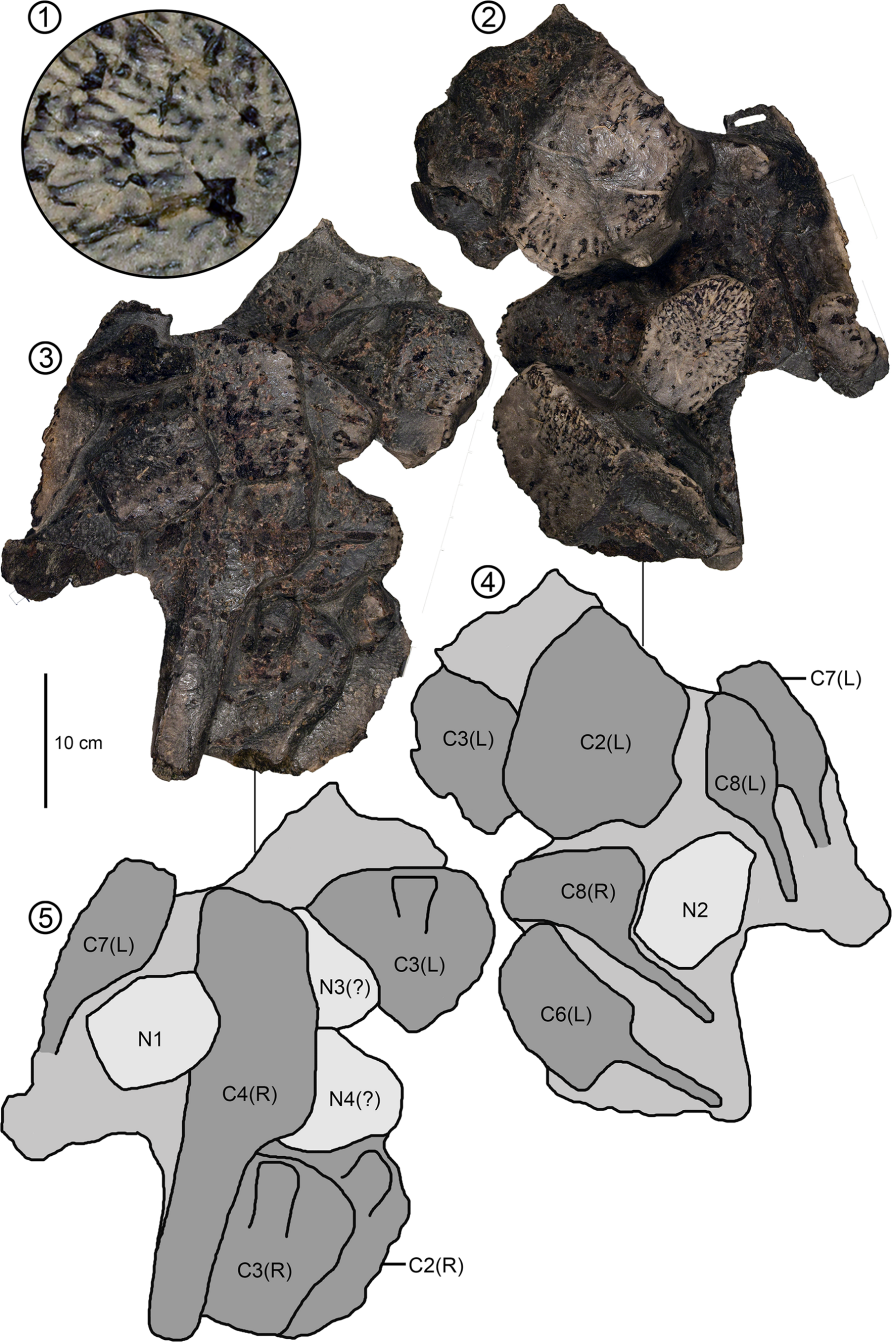
*Peritresius ornatus*, slab specimen, ALMNH 8988 from the upper Campanian of Alabama, USA. (1) 10X magnified view of dermal sculpturing present on all preserved elements. (2, 4) slab in dorsal view. (3, 5) slab in ventral view. Abbreviations: C, costal; N, neural.

**Fig 7:**
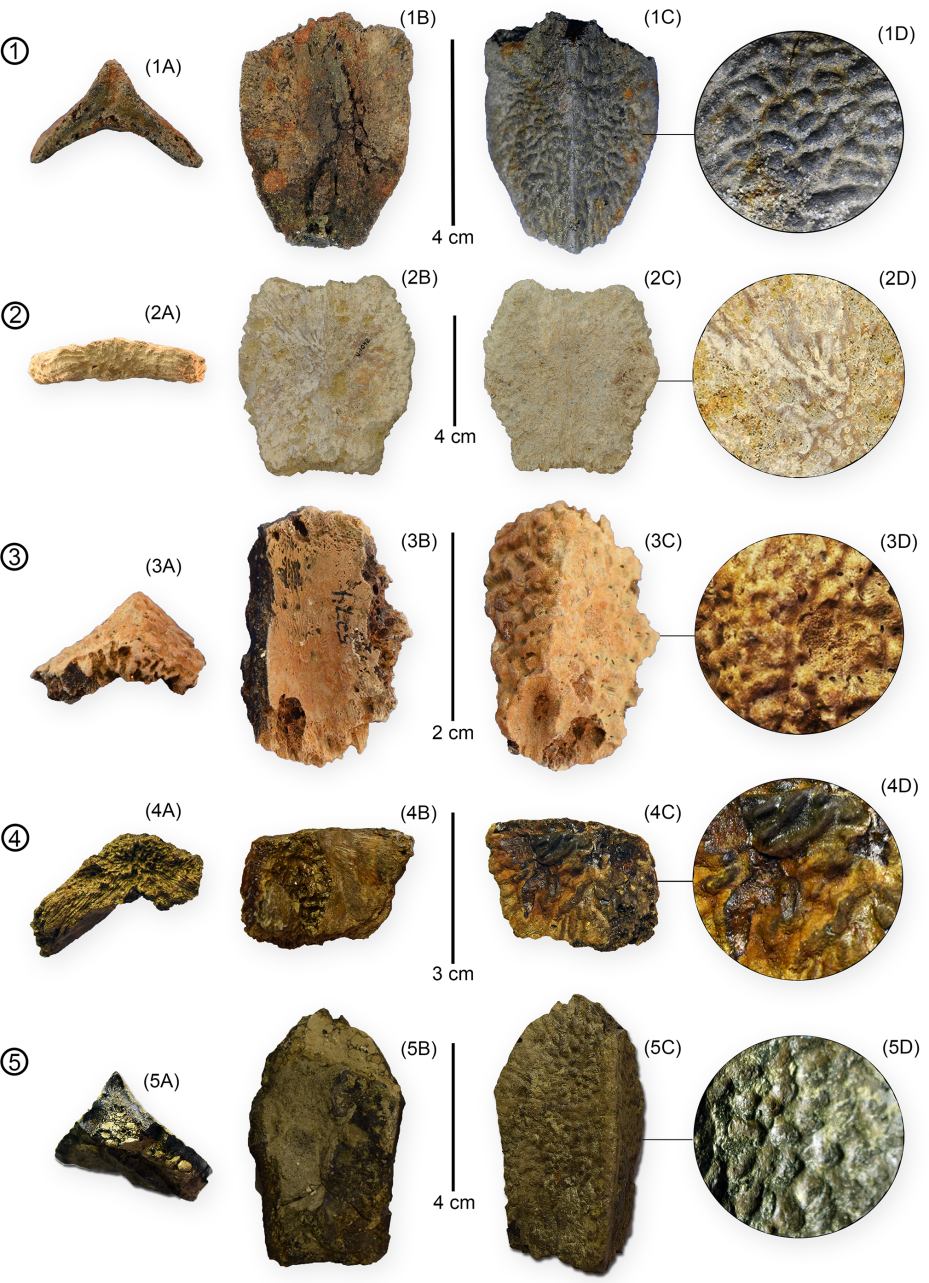
*Peritresius ornatus* neurals from the Campanian-Maastrichtian of Alabama and Mississippi. (1) MMNS 5876; (2) ALMNH 5497; (3) MMNS 5274; (4) MMNS 5710; (5) MMNS 8632.4. All elements shown in (A) anterior, (B) ventral, (C) dorsal, and (D) 10X magnified views.

**Fig 8:**
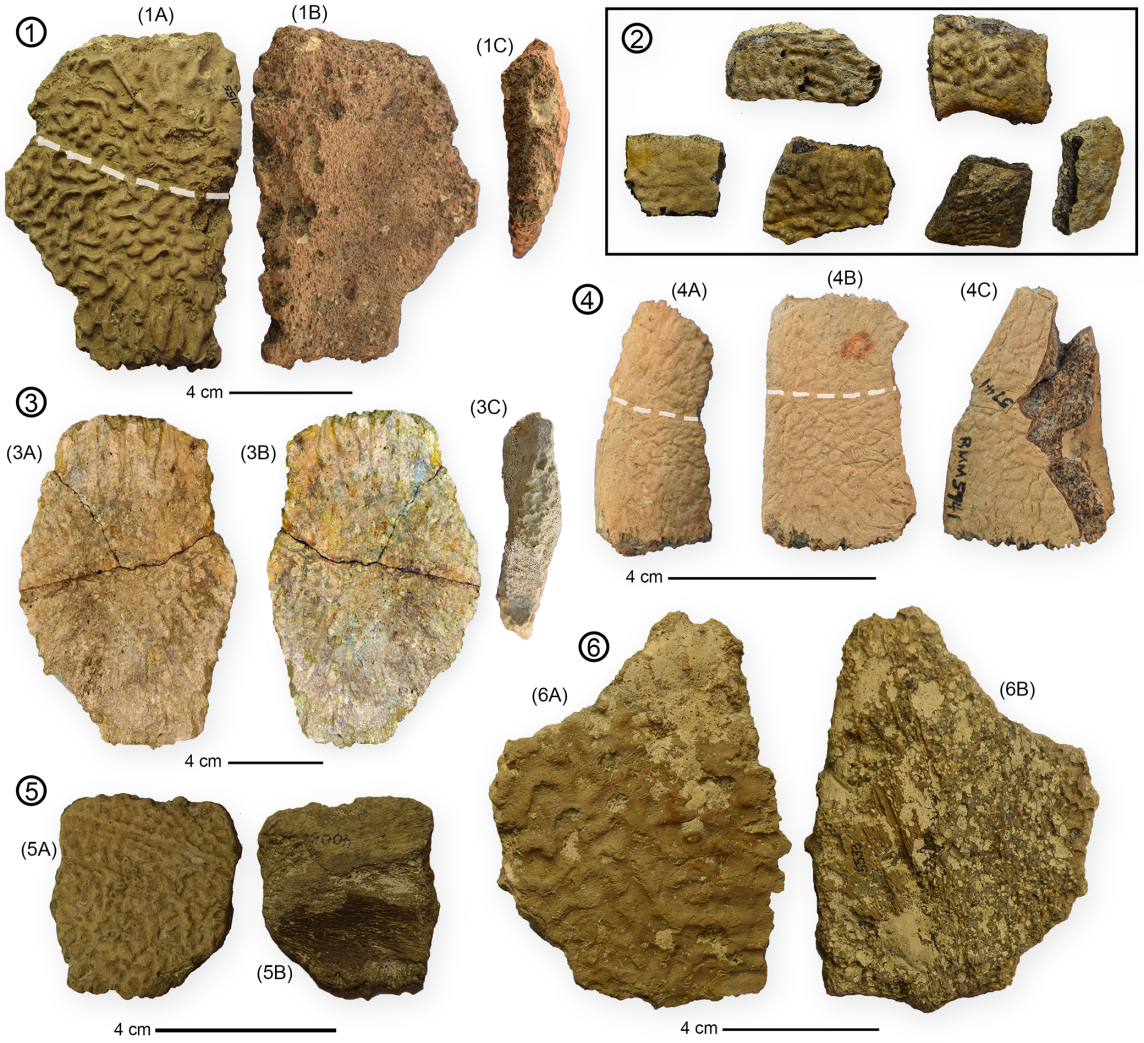
*Peritresius ornatus* peripheral and costal material from Alabama and Mississippi. (1) MMNS 4547 in: (A) dorsal, (B) ventral, and (C) posterior views; (2) MMNS 4003 in dorsal view; (3) ALMNH 6256 in: (A) dorsal, (B) ventral, and (C) posterior views; (4) RMM 5741 in dorsal view; (5) MMNS 5102 in: (A) dorsal and (B) ventral views; (6) MMNS 5533 in: (A) dorsal and (B) ventral views.

1856 *Chelone ornata* Leidy [10]: 105, pl. 18, fig 10.

1869 *Peribresius* [sic, errore] *ornatus*. Cope *in* Cook [50]: 735.

1869 *Peritresius ornatus* Cope [51]: 88; 1870: 150.

1870 *Prochonias nodosus* Cope [52]: 158, 159.

1870 *Taphrosphys nodosus* Cope [52]: 167, 244, pl. 1, fig 16.

1908 *Peritresius ornatus = ? Taphrosphys nodosus* Hay [31]: 122, 210.

1955 *Peritresias* [sic] *ornatus* Miller [53]: 908.

1964 *Peritresius ornatus* Baird [4]

### Referred Specimens

ALMNH 3900 –Various highly fragmented carapacial and plastral elements. ALMNH 3780 –Plastral fragment. ALMNH 5497 –Nearly complete neural. ALMNH 5887 –Partial left costal missing distal rib-end, right hypoplastron, anterior half of one neural, and several peripheral/costal fragments. ALMNH 6256 –Carapace fragments including the dorsal face of an anterior peripheral (4–6?). ALMNH 8988 –Several dissociated costals and four anterior neurals. AMNH 1410 –Isolated carapacial scraps. AMNH 1480 –Two partial costals, an anterior neural, and numerous carapacial fragments. MMNS 4003 –Costal fragment. MMNS 4407 –Costal fragment. MMNS 4546 –Partial neural. MMNS 4547 –Single peripheral. MMNS 5102 –Pieces of several costals. MMNS 5274 –Partial neural (juvenile?). MMNS 5533 –Large costal fragment. MMNS 5710 –Partial neural. MMNS 8632.4 –Single neural of a large individual; MSC 5741 –Small individual; three articulated anterior peripherals, possibly peripherals 3–5 of the left side.

### Description of new material

#### Carapace

Though none of the newly identified specimens of *P*. *ornatus* possess an intact carapace, the Alabama and Mississippi material can be assigned to *P*. *ornatus* based on the presence of pronounced vermiculate sculpturing consisting of irregular grooves and channels located on the outer surfaces of all costals, peripherals, and neurals (Figs 6–8). The seemingly random distribution of papillae and rugosities formed by the sculpturing found on these specimens differs greatly from the pitting found on the surfaces of the dermal elements of trionychid turtles, but appears identical to that described on *P*. *ornatus* (see [4]).

#### Costals

Though numerous costal pieces can be identified from among the specimens in our sample, there is very little diagnostic information that can be derived from many of these elements due to their high degree of fragmentation. However, one specimen (ALMNH 8988) possesses six nearly intact costals and the medial portions of four others (Fig 6). The degree to which the costal plates extend laterally along the length of each element and the sizes of the resulting fontanelles created between the distal rib-ends of each costal pair appear identical to those of the holotype ([4], figs. 12 and 13). The carapacial fontanelles of *P*. *ornatus* are pronounced, even in large, presumably adult, individuals (such as ALMNH 8988) and are more similar to those of *P*. *martini* and *A*. *hofmanni* than to other well-described Cretaceous pan-cheloniids such as *Ctenochelys stenoporus*. The robust nature of these elements is also noteworthy with the anterior costals of ALMNH 8988 being nearly 3 cm thick, owing primarily to the dense layer of cortical bone covering the surfaces of each element.

#### Neurals

Although the preserved neurals exhibit a considerable amount of variation in overall size, each possesses the distinct deep sculpturing inherent to carapacial material belonging to *P*. *ornatus* (Fig 7). The largest of the neurals is slightly wider (7.9 cm) than long (7.4 cm), generally hexagonal, and despite being preserved with significant dorsoventral compression, retains a mid-dorsal keel indicative of ‘lophechelyine’-grade taxa such as *Prionochelys* and *Ctenochelys* (ch.125/1).

The smallest neural in our sample, which measures 2.5 cm in length and 1.8 cm in width, is interpreted here as representing the first juvenile material recovered for this taxon (Fig 7.3). The relative dimensions of this element implies that the neurals of *P*. *ornatus* increased more in width than in length as the turtle matured. The posterior third of the mid-dorsal keel of this neural is excavated into an ovoid depression, presumably the insertion point for the first epineural, indicating that this is neural 3. The neurals of *P*. *ornatus* are relatively thicker than those of *Ctenochelys* with a gradual decrease in thickness from the anterior to posterior positions.

### Peripherals

The peripherals of *P*. *ornatus* are generally longer than wide and possess a moderate lateral serration (Fig 8). Due to the isolated nature of the peripherals described here, very little can be ascertained regarding the total number of these elements, or their position, relative to the remainder of the carapace.

## Discussion

### Phylogenetic placement of *Peritresius*

**Fig 9:**
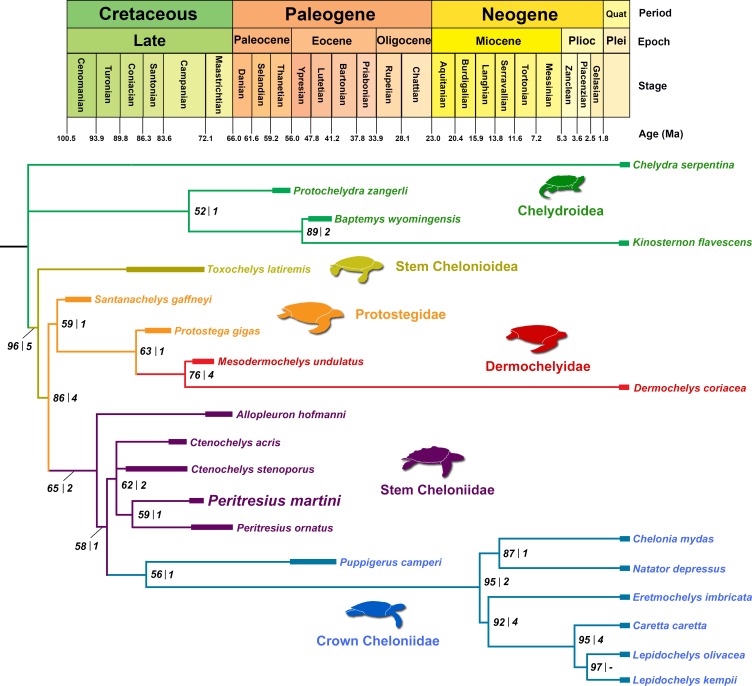
Time-calibrated, strict consensus phylogeny of select fossil and extant Testudine species. Bootstrap values (*left*) and decay indices (*right*) are shown for each node; CI = 0.586; RI = 0.671; Branch lengths for crown cheloniid species taken from Cadena and Parham [28].

Phylogenetic analyses resulted in 3 equally parsimonious trees with a length of 307 steps. The strict consensus tree places *Peritresius* spp. within Pan-Cheloniidae as a sister group to *Ctenochelys* spp. and *A*. *hofmanni* (Fig 9). The hypothetical sister relationship between *Ctenochelys* and *Peritresius*, first proposed by Baird [4] and again supported by Hirayama [1] based on the presence of epineurals, is also seen here based on additional postcranial characters (see below). Although epineurals have also been observed in other species of fossil marine turtle (e.g. *Archelon ischyros* Wieland 1896 [54]), and as an ontogenetically variable characteristic in the extant cheloniid *Lepidochelys kempii* (Garman 1880 [55]), the general arrangement of these elements in *Peritresius* more closely resembles the neural-epineural conformation of *Ctenochelys* than those of any other fossil or extant marine turtle. Hirayama’s [1] monophyletic grouping of *Ctenochelys* and *Peritresius* also included species belonging to the North American genus *Prionochelys*, but due to a lack of described material for members of this genus and the resulting confusion surrounding their taxonomy, no species of *Prionochelys* could be adequately incorporated into the present matrix.

The present study did not recover a single unambiguous postcranial synapomorphy uniting the species of *Peritresius*, and only one unambiguous postcranial synapomorphy was identified for Pan-Cheloniidae which is the presence of a rib-free peripheral between the ribs of the 7th and 8th costals (ch. 133/2). *Peritresius* spp. is grouped with *Ctenochelys* spp. and *A*. *hofmanni* based on the following characteristics: medial line of keels on the dorsal surface of the carapace (ch. 116/3), posteromedial nuchal fontanelles (ch. 123/1), and an extreme reduction in lateral costal ossification resulting in the dorsal exposure of the distal rib ends in almost every costal series (ch. 132/3). All of these characters are found in other chelonioids. The shared character states among distinct lineages highlights the homoplastic morphology of chelonioids and the importance of additional descriptions of Cretaceous chelonioid specimens, especially those possessing both cranial and postcranial elements.

### Biostratigraphy and paleobiogeography of Late Cretaceous chelonioids *sensu stricto*

**Fig 10:**
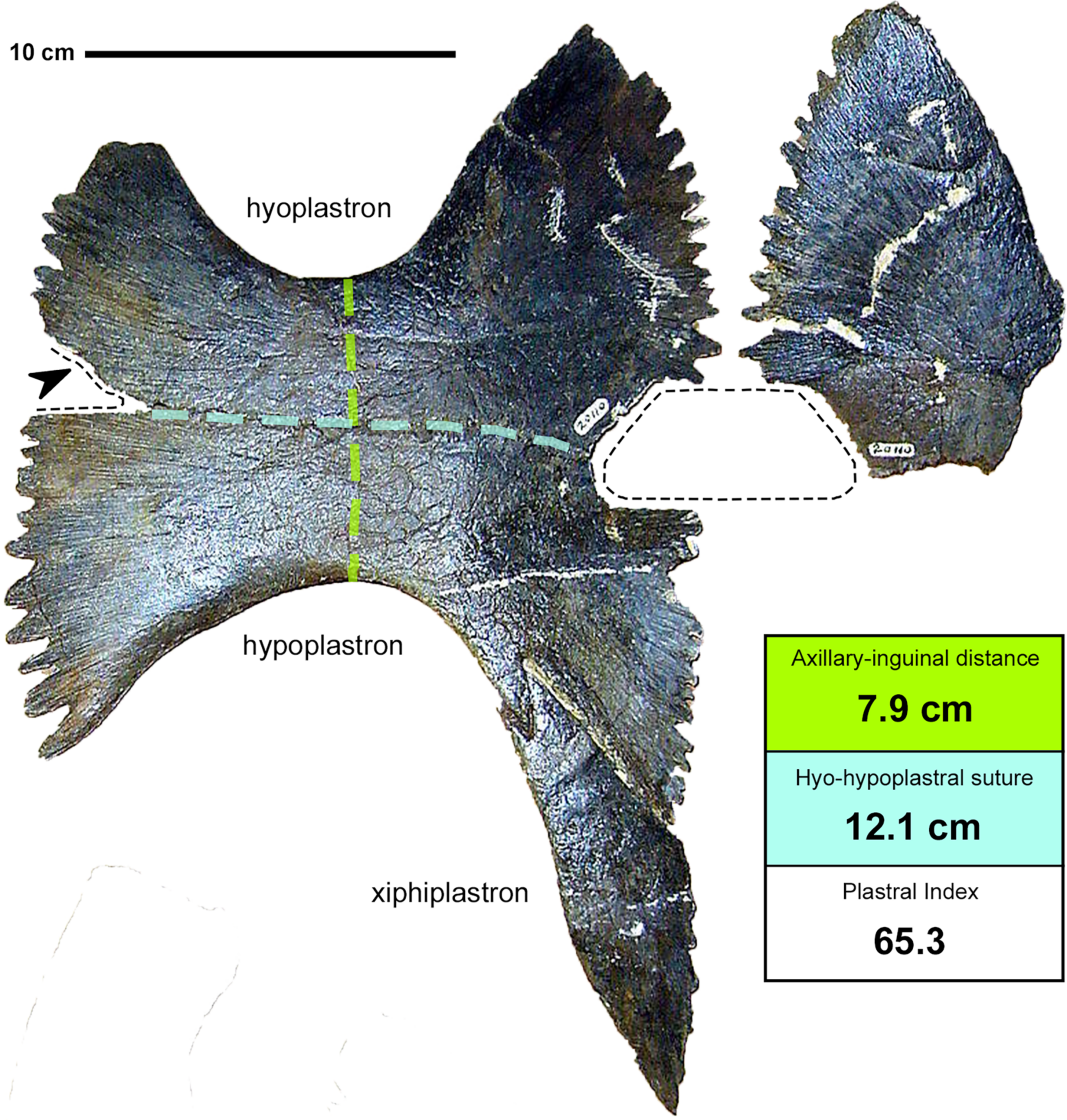
*Toxochelys weeksi*, plastron, USNM.V.20110 (holotype) from the upper Campanian of Tennessee. Plastron in ventral view with associated measurements. Dashed lines represent areas historically interpreted as fontanelles and the black arrow indicates a broken edge. Courtesy of Smithsonian Institution. Photograph by A. Millhouse.

The discovery of *Peritresius* remains from the Campanian of Alabama closes the temporal gap noted by Baird [4] between *Peritresius* and other ‘toxochelyid’-grade taxa and makes *Peritresius* the only Cretaceous pan-chelonioid genus known to cross the Campanian-Maastrichtian boundary [2,56]. Several authors have proposed the continuation of the *Toxochelys latiremis* lineage from the Campanian into the Maastrichtian [1,57] based on the synonymy of *T*. *latiremis* and *Toxochelys weeksi* Collins, 1951 [58] from the Ripley Formation of Tennessee. This synonymy was originally proposed by Nicholls [59] based on an expansion in the accepted range of intraspecific variability with regard to proportions of the plastron. *T*. *weeksi* is represented only by the holotype (USNM.V.20110—previously UT K20) which consists of a partial plastron and three associated posterior peripherals. This specimen was illustrated by both Collins [58] and Zangerl [2], however neither author provided photographs of the specimen. Nicholls [59] noted in her taxonomic reassessment of *Toxochelys latiremis* that the holotype of *Toxochelys weeksi* was the only specimen she did not personally examine. Recently, photographs of the holotype of *Toxochelys weeksi* were made available online by the USNM Department of Paleobiology which show that the precise size of at least the lateral plastral fontanelle of this specimen is impossible to adequately determine due to missing pieces of bone at the posterolateral margin of the hyoplastron (Fig 10).

Nicholls [59] accurately notes that the plastral measurements reported by Zangerl ([2], p. 174) for this specimen are wrong and once the correct measurements are taken (Fig 10), the values much more closely align with those of the holotype of *T*. *moorevillensis* (FMNH 27330) than to any referred specimen of *T*. *latiremis*. The xiphiplastron of USNM.V.20110 also more closely resembles the xiphiplastron of *T*. *moorevillensis* than that of *T*. *latiremis*. Additionally, the Coon Creek Tongue of the Ripley Formation of Tennessee from which USNM.V.20110 was recovered, previously thought to be early Maastrichtian in age, has more recently been interpreted to fall within the latest Campanian [60]. Given the questionable morphology and horizon of USNM.V.20110, *T*. *weeksi* should no longer be considered a junior synonym of *T*. *latiremis* and is here referred to *T*. *moorevillensis*. The youngest specimens of *T*. *latiremis* are herein considered to be those from the Late Campanian Pierre Shale of Kansas and South Dakota.

The presence of *Peritresius* in the Maastrichtian of the northeastern Atlantic Coast (i.e. New Jersey and Maryland) makes it just the third Cretaceous pan-cheloniid known from this area (along with *Euclastes* and *Catapleura*). A large humerus belonging to the Campanian marine turtle *Atlantochelys mortoni* Agassiz, 1849 [61] was recovered from the Mount Laurel Formation of New Jersey (ANSP 9234) but given that the species is known from a single element, it is impossible to generate a large enough suite of characters to adequately incorporate *A*. *mortoni* into a phylogenetic analysis. As a result, its cladistic affinities cannot be determined with any confidence. Based solely on humeral morphology, *A*. *mortoni* has been hypothesized as a member of either Protostegidae [1,62] or Cheloniidae [63] but until more material of this species is recovered and the species’ phylogenetic placement formally tested, we conservatively exclude this specimen from any discussions pertaining to the cladistics or paleobiogeography of Cretaceous chelonioids *sensu stricto* given the possibility that *A*. *mortoni* may not belong to this clade.

**Fig 11:**
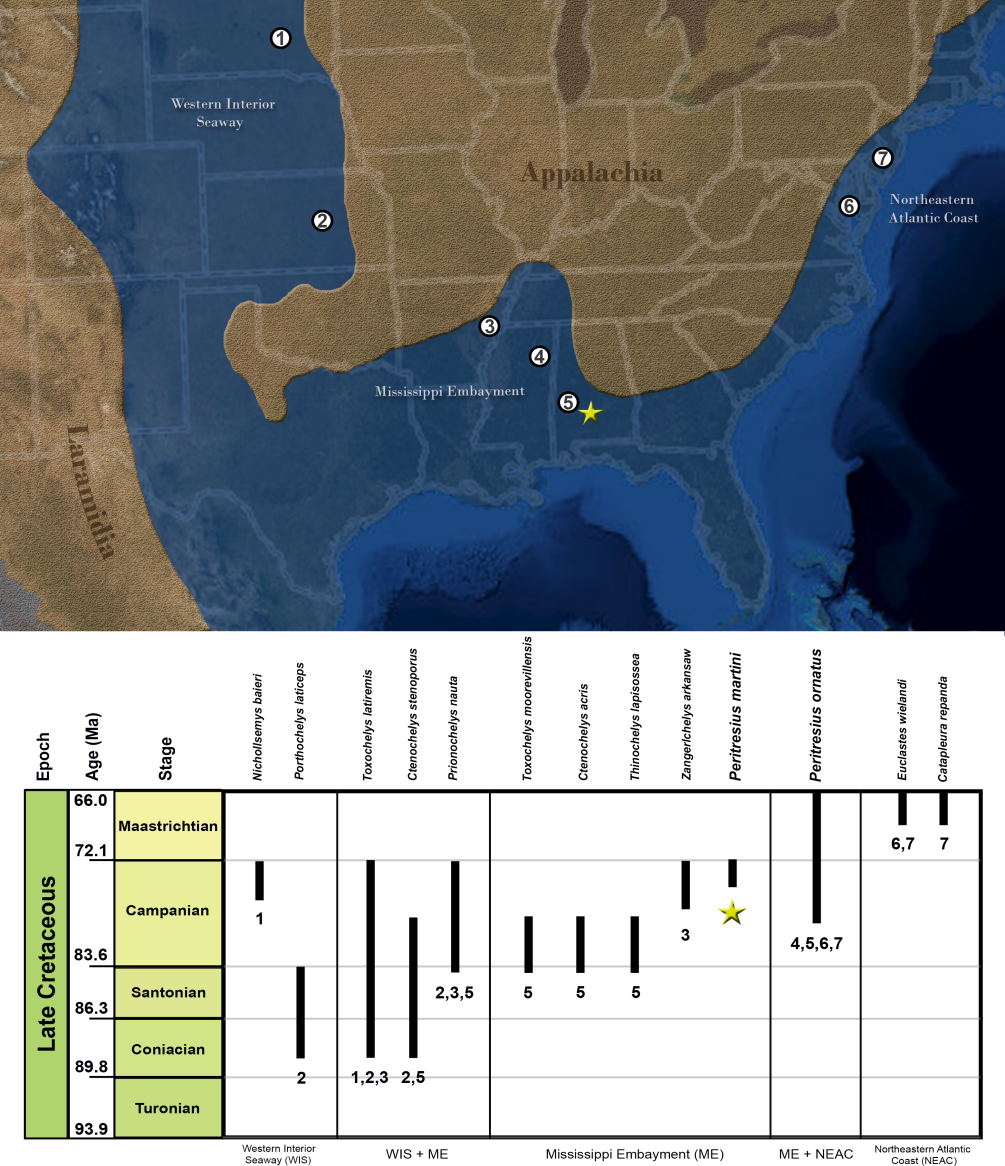
Biostratigraphy and paleobiogeography of Late Cretaceous chelonioid *sensus stricto* species of North America. Localities and taxon ranges for fossil occurrences were taken from the literature as follows: *Nichollsemys baieri* from Brinkman et al. [64], *Porthochelys laticeps* from Hirayama [1], *Toxochelys latiremis* from Hirayama [1] and this study, *Ctenochelys stenoporus* from Hirayama [1], *Prionochelys nauta* from Hirayama [1], *Toxochelys moorevillensis* from Hirayama [1], *Ctenochelys acris* from Gentry [29], *Thinochelys lapisossea* from Hirayama [1], *Zangerlchelys arkansaw* from Hirayama [1], *Peritresius martini* from this study, *Peritresius ornatus* from Baird [4] and this study, *Euclastes wielandi* from Parham [65], and *Catapleura repanda* from Hirayama [66]. Age justifications are provided as supporting information (S4 File). Base map obtained and modified from the USGS National Map Viewer.

The *Peritreus* material described herein makes this genus the only late Cretaceous marine turtle known from both the Mississippi Embayment and the northeastern Atlantic Coast despite the prevalence of marine turtle fossils in both areas. Based on the fossil material currently known for the genus, it seems that the unsculptured species of *Peritresius* (*P*. *martini*) did not disperse beyond the Mississippi Embayment and unlike the predominantly Maastrichtian *P*. *ornatus*, is known exclusively from Campanian deposits (Fig 11). The limited distribution of *P*. *martini* fits the previously noted pattern of endemic speciation common among Cretaceous chelonioids *sensu stricto* [1].

### Sculptured vs. unsculptured *Peritresius*

The existence of an unsculptured species of *Peritresius* (*P*. *martini*.) makes *Peritresius* the first genus of marine turtle to contain both a sculptured and unsculptured form. The irregular, dermal sculpturing of *P*. *ornatus* differs from that of trionychids and adocids in that there is no discernible pattern in the arrangement of the ridges and papillae that form the sculpturing, other than the occasional appearance of brief channels created by the alignment of seemingly random dorsally protruding trabeculae (Figs 6–8). The vermiculate sculpturing of *P*. *ornatus* seems to more closely resemble the condition observed in the Eocene cheloniid *Osonachelus decorata* de Lapparent de Broin, Murelaga, Farres, and Altimiras 2014 [67], the Oligocene cheloniids *Ashleychelys palmeri* Weems and Sanders, 2014 [42] and *Carolinochelys wilsoni*, and the Neogene pan-cheloniid *Trachyaspis lardyi* Meyer 1843 [68] (= *Syllomus aegyptiacus* [Lydekker 1889 [69]] fide Villa and Raineri 2015 [70]).

Though the morphology of the sculpturing found on certain fossil marine turtles has received some attention [4,67,70,71], little effort has yet been made to identify potential functions of this feature. Any hypothesis regarding the functional basis of the dermal sculpturing of fossil marine turtles would have to rely heavily upon inferences from modern taxa, but unfortunately, even though slight carapacial sculpturing has been described in *Natator depressus* Garman, 1880 [55] (see [72]), no extant cheloniid exhibits the high degree of ornamentation found in *P*. *ornatus*. Several groups of non-marine turtles are ornamented, including various pan-trionychians, all solemydids, and most pleurosternids. The shell histology of these taxa is well documented [73–76] but we are not aware of any published studies about the function of the shell sculpturing. However, highly ornamented, irregular sculpturing of the dermal elements is present among other animals, such as squamates [77–79] and basal tetrapods [80], where this feature is the result of highly vascularized bones necessary for thermal regulation via the alteration of blood flow to and from the dermis.

*Peritresius ornatus* is the only Campanian marine turtle known to persist from the early Campanian into the Maastrichtian (Fig 11), an interval where there is strong isotopic evidence that ocean temperatures were dropping [81,82]. This cooling and other environmental perturbations have already been plausibly linked to a dip in the diversity of large marine tetrapods during this time (*e*.*g*. mosasaurs; [83]) and may also have been responsible for the apparent drop in chelonioid diversity during the Maastrichtian [1,6]. If shell sculpturing does relate to thermoregulation, then it is possible that the persistence of *P*. *ornatus* into the cooler Maastrichtian may have been facilitated by this feature. If so this would be one of the few examples of fossilized characters relating to thermoregulation in marine turtles (e.g. [84]).

## Conclusions

A new species of Cretaceous marine turtle from the southeastern United States (*Peritresius martini* sp. nov.) is herein described based on material collected from the upper Campanian of Alabama, USA. *Peritresius martini* sp. nov. differs from *Peritresius ornatus* in having a ‘rib-free’ 10th peripheral, a less pronounced lateral keel on the anterior peripherals, and an unsculptured carapace and plastron. The heavily vascularized and sculptured dermal elements characteristic of *P*. *ornatus* are interpreted here as potentially indicative of a thermoregulatory capability and may have been one of the key factors contributing to the survival of *Peritresius* into the Maastrichtian, a period of cooling when other lineages of Campanian marine turtles (e.g., Protostegids, *Toxochelys*, and *Ctenochelys*) went extinct.

## Supporting information

S1 File Character-taxon matrix in Mesquite format. (NEX)

S2 File Character list. (PDF)

S3 File Molecular scaffold. (JPG)

S4 File Fig 11 supporting information. Including (I) Locality age estimates; (II) Literature cited.
(PDF)

## Abbreviations

ALMNH: Alabama Museum of Natural History, Tuscaloosa, USA
AMNH: American Museum of Natural History, New York City, NY, USA
ANSP: Academy of Natural Sciences of Drexel University, Philadelphia, PA, USA
ChM: Charleston Museum, Charleston, SC, USA
MMNS: Mississippi Museum of Natural Sciences, Jackson, USA
MSC: McWane Science Center, Birmingham, AL, USA
NJSM: New Jersey State Museum, Trenton, NJ, USA
USNM: United States National Museum, Washington D.C., USA
