# Supplementary material for: A new species of Peritresius Leidy, 1856 (Testudines: Pan-Cheloniidae) from the Late Cretaceous (Campanian) of Alabama, USA, and the occurrence of the genus within the Mississippi Embayment of North America
Source: PLoS One. 2018 Apr 18;13(4):e0195651. doi: 10.1371/journal.pone.0195651 (PMC5906092; doi:10.1371/journal.pone.0195651)
Supplement: S2 File — (PDF) [file pone.0195651.s002.pdf]

Character list

List of characters used in phylogenetic analyses. Authors abbreviations: CP, Cadena & Parham, (2015). See respective reference for discussion, character history, synonymies, and all previous references.

Skull

1. Nasals: 0 = present; 1 = absent. CP (ch 1)
2. Nasals, medial contact of nasals: 0 = nasals contact one another medially along their entire length; 1 = medial contact of nasals partially or fully hindered by long anterior frontal process. CP (ch 2).
3. Nasals, size of nasals: 0 = dorsal exposure of nasals large; 1 = dorsal exposure of nasals greatly reduced relative to that of the frontals. CP (ch 3).
4. Prefrontals, medial contact of prefrontals on the dorsal skull surface: 0 = absent; 1 = present, absence of contact between the nasal or apertura narium externa and the frontal. CP (ch 4).
5. Prefrontals, prefrontal-vomer contact: 0 = present; 1 = absent. CP (ch 5).
6. Prefrontals, prefrontal-palatine contact: 0 = present; 1 = absent. CP (ch 6).
7. Prefrontals, dorsal prefrontal exposure: 0 = present, large; 1 = reduced; 2 = absent or near absent. CP (ch 7). Ordered.
8. Prefrontals, cranial scutes on the prefrontal: 0 = one pair; 1 = two pairs or more. CP (ch 8).
9. Lacrimal: 0 = present; 1 = absent. CP (ch 9).
10. Frontals, frontal contribution to orbit: 0 = absent, contact between prefrontal and postorbital; 1 = present. CP (ch 10).
11. Frontals, both frontals medially fused: 0 = absent; 1 = present. CP (ch 11).
12. Frontals, direction of the orbits in dorsal view of the skull: 0 = laterally facing, with a very narrow to almost complete absent dorsal exposure of the maxilla and jugal; 1 = dorsolateral facing, with portions of the maxilla and jugal dorsally exposed. CP (ch 12).
13. Parietals, parietal-squamosal contact: 0 = present, upper temporal emargination absent or poorly developed; 1 = absent, upper temporal emargination well developed. CP (ch 13).
14. Parietals, closure of foramen nervi trigemini and the length of the anterior extension of the lateral braincase wall: 0 = foramen nervi trigemini anteriorly open, anterior extension of lateral braincase wall absent; 1 = foramen nervi trigemini anteriorly closed, processus inferior parietalis only produces a narrow strut anterior to the foramen nervi trigemini, usually absence of contact with palatine; 2 = foramen nerivi trigemini anteriorly closed, processus inferior parietalis produces an extended process anterior to the foramen nervi trigemini, contact with palatine commonly present. CP (ch 14).
15. Parietals, posterodorsal margin of the temporal fossa roofed by an overhanging process of the skull roof: 0 = absent; 1 = present. CP (ch 15).
16. Parietals, contribution to the processus trochlearis oticum: 0 = absent; 1 = present. CP (ch 16).
17. Parietals, foramen stapedio-temporalis: 0 = absent or weak, foramen stapedio-temporale concealed in dorsal view; 1 = moderate foramen stapedio-temporale, partial exposition of the processes trochlearis in dorsal view; 2 = strong, entire exposition of the processus trochlearis in dorsal view. CP (ch 17). Ordered.
18. Parietals, pineal foramen located medially between parietals: 0 = absent; 1 = present. CP (ch 18).

19. Jugals, jugal-squamosal contact: 0 = present; 1 = absent, contact between postorbital and quadratojugal present. CP (ch 19).
20. Jugals, jugal participation in the rim of the upper temporal emargination: 0 = absent; 1 = present, upper temporal emargination extensive. CP (ch 20).
21. Jugals, jugal-quadrate contact: 0= absent; 1= present, quadratojugal does not contribute to lower temporal margin. CP (ch 21).
22. Jugals, medial process of jugal beneath orbit, seen in ventral view to slightly ventroposterior view: 0 = weakly developed or absent, jugal only contacts the maxilla; 1 = weak to moderately developed, presence of a contact between the jugal and pteygoid due to the lateral extension of this last; 2 = strongly developed, jugal contacts the pterygoid, the palatine, and the maxilla. CP (ch 22). Ordered.
23. Quadratojugals, deep lower temporal emargination extending above the upper limit of the cavum tympani and the resulting loss of the quadratojugal: 0 = absent; 1 = present. CP (ch 23).
24. Quadratojugals, quadratojugal-maxilla contact: 0 = absent; 1 = present, jugal does not contribute to lower temporal emargination. CP (ch 24).
25. Quadratojugals, quadratojugal-squamosal contact below the cavum tympani: 0 = absent; 1 = present. CP (ch 25).
26. Squamosals, squamosal-postorbital contact: 0 = present; 1 = absent, temporal roofing well developed, but postorbital short; 2 = absent, due to lower temporal emargination; 3 = absent, due to upper temporal emargination. CP (ch 26).
27. Squamosals, squamosal-supraoccipital contact: 0 = absent; 1 = present. CP (ch 27).
28. Squamosals, posterolateral protuberances developing horns: 0 = absent; 1 = present. CP (ch 28).
29. Squamosals, very long posterior process, formed exclusively by the squamosal and protruding beyond condyles occipitalis: 0 = absent; 1 = present. CP (ch 29).
30. Squamosals, squamosal-quadrate contact: 0 = tightly sutured; 1 = wide open. CP (ch 30).
31. Postorbitals, postorbital-palatine contact: 0 = absent; 1 = present, foramen palatinum posterius situated posterior to the orbital wall. CP (ch 31).
32. Supratemporal: 0 = present; 1 = absent. CP (ch 32).
33. Premaxilla, subdivision of the apertura narium externa by an internarial process of the premaxilla only: 0 = present; 1 = absent. CP (ch 33).
34. Premaxilla, fusion of premaxillae: 0 = absent; 1 = present. CP (ch 34).
35. Premaxilla, foramen praepalatinum: 0 = present; 1 = absent; 2 = absent, foramen intermaxillaris present. CP (ch 35).
36. Premaxilla, exclusion of the premaxillae from the apertura narium externa: 0 = absent; 1 = present. CP (ch 36).
37. Premaxilla, distinct, medial premaxillary hook along the labial margin of the premaxillae: 0 = absent; 1 = present. CP (ch 37).
38. Palatines, palatine contribution to the anterior extension of the lateral braincase wall: 0 = absent; 1 = present, well-developed. CP (ch 38).
39. Palatines, contribution to the upper triturating surface: 0 = absent or less than 30% of the total width of the triturating surface; 1 = present, at least 30% or more of the total width of the triturating surface. CP (ch 39).
40. Palatines, secondary palate: 0 = absent; 1 = present, complete separation of the narial cavity from the oral cavity. CP (ch 40).

41. Palatines, vomer-palatine contact anterior to internal naris (apertura narium interna): 0 = absent; 1 = present. CP (ch 41).
42. Maxilla, triturating surface definition: 0 = triturating surface with labial ridge only; 1 = triturating surface with labial and lingual ridge; 2 = triturating surface with labial, lingual, and accessory ridge(s). CP (ch 42). Ordered.
43. Maxilla, accessory ridge(s): 0 = accessory ridge(s) on maxilla present along the triturating surface; 1 = accessory ridge(s) only in some sectors of the triturating surface. CP (ch 43).
44. Vomer, number of vomer(s): 0 = paired; 1 = single, but large; 2 = single and greatly reduced or absent. CP (ch 44).
45. Vomer, vomer-pterygoid contact in palatal view: 0 = present; 1 = absent, medial contact of palatines present. CP (ch 45).
46. Vomer, vomerine and palatine teeth: 0 = present; 1 = absent. CP (ch 46).
47. Vomer, vomer-premaxilla contact in ventral view: 0 = broad, anterior margin of the vomer straight; 1 = very reduced, anterior margin of vomer forming an acute tip; 2 = absent, both maxilla meeting medially. CP (ch 47). Ordered.
48. Vomer, ventral crest: 0 = absent; 1 = narrow and tall ventral crest present all along the vomer. CP (ch 48).
49. Vomer, shape of the palate roof: 0 = flat; 1 = domed. CP (ch 49).
50. Vomer, vomerine pillar visible in ventral view: 0 = vomerine pillar absent; 1 = present; 2 = present but obscured in ventral view by the posterior extension of the triturating surface of the vomer. CP (ch 50). Ordered.
51. Vomer, contribution to the upper triturating surface; 0 = absent, triturating surface narrow to absent; 1 = present. CP (ch 51).
52. Quadrates, flooring of cavum acustico-jugulare and recessus scale tympani: 0 = absent; 1 = fully or partially present, produced by the posterior process of the pterygoid, but the pterygoid does not cover the prootic; 2 = produced by the posterior process of the pterygoid, and the pterygoid covers the prootic; 3 = fully or partially present, produced by the ventral process of the quadrate or the prootic, or both. CP (ch 52).
53. Quadrates, development of the cavum tympani: 0 = shallow, but not developed anteroposteriorly; 1 = shallow, but anteroposteriorly developed; 2 = deep and anteroposteriorly developed. CP (ch 53). Ordered.
54. Quadrates, precolumellar fossa: 0 = absent; 1 = present. CP (ch 54).
55. Quadrates, antrum postoticum: 0 = absent; 1 = present, quadrate does not fully enclose the anterior perimeter of the antrum; 2 = present, quadrate fully encloses the anterior perimeter of the antrum. CP (ch 55). Ordered.
56. Quadrates, arrangement between the quadrate, ophisthotic, stapes and Eustachian tube: 0 = the quadrate and the opisthotic form an angle of 90 degrees in lateral view; 1 = present, but the quadrate and the opisthotic form an angle less than 90 degrees in lateral view; 2 = the quadrate is well developed posteroventrally enclosing only the stapes; 3 = the quadrate is well developed posteroventrally enclosing the stapes and the Eustachian tube; 4 = the quadrate enclosing stapes and the Eustachian tube helped by the posteroventral projection of the squamosal and posterior of the quadratojugal. CP (ch 56).
57. Quadrate, processus trochlearis oticum: 0 = absent; 1 = present, very reduce; 2 = present, large forming a well-defined musculatory facet. CP (ch 57). Ordered.

58. Quadrate, contribution to the musculatory facet of the processus trochlearis oticum: 0 = extensive contribution; 1 = small contribution, facet formed principally by the protic and/or parietal. CP (ch 58).
59. Quadrate, qudrate-basiphenoid contact: 0 = absent; 1 = present. CP (ch 59).
60. Epipterygoids: 0 = present, rod like; 1 = present, laminar; 2 = absent. CP (ch 60).
61. Pterygoids, pterygoid teeth: 0 = present; 1 = absent. CP (ch 61).
62. Pterygoids, basipterygoid process and basipterygoid articulation: 0 = basipterygoid process present with a movable basiptergoid articulation; 1 = basipterygoid process present with a sutured basipterygoid articulation; 2 = basipterygoid process absent and sutured basipterygoid articulation. CP (ch 62).
63. Pterygoids, interpterygoid vacuity: 0 = triangular in shape; 1 = reduced to an interpterygoid slit; 2 = reduced to a paired foramen anterius canalis carotici palatinum. Modified from CP (ch 63). Ordered.
64. Pterygoids, pterygoid-basioccipital contact: 0 = absent; 1 = present. CP (ch 64).
65. Pterygoids, processus trochelaris pterygoideus: 0 = absent; 1 = present. CP (ch 65).
66. Pterygoids, foramen palatinum posterius: 0 = present; 1 = present, but open laterally; 2 = absent. CP (ch 66). Ordered.
67. Pterygoids, medial contact of pterygoid: 0 = present, pterygoids in a very long medial contact with one another, longer than the basisphenoid total length in midline; 1 = present, pterygoids in medial contact with one another, contact length equal or shorter than the basisphenoid total length in midline; 2 = absent, contact of the basisphenoid with the vomer and/or palatines present. CP (ch 67) Ordered.
68. Pterygoids, pterygoid contribution to foramen palatinum posterius: 0 = present; 1 = absent. CP (ch 68).
69. Pterygoids, vertical flange on processus pterygoideus externus: 0 = absent; 1 = present. CP (ch 69).
70. Pterygoids, contact with the exoccipital: 0 = absent; 1 = present. CP (ch 70).
71. Pterygoids, fossa podocnemidoidea or cavum pterygoidei: 0 = absent; 1 = present. CP (ch 71).
72. Pterygoids, processus pterygoideus externus: 0 = large, forming an extensive lateral wing; 1 = reduced, forming an acute tip; 2 = extremely reduced due to the posterolateral projection of the pterygoid; 3 = absent. CP (ch 72). Ordered.
73. Pterygoids, level of the position of the pterygoid respect to basisphenoid: 0 = both bones are at the same level on ventral surface; 1 = two different levels, creating a step between the two bones. CP (ch 73).
74. Pterygoids, medial ridge: 0 = incipient to absent; 1 = present, ridge spans nearly the full length of the pteygoids, sometimes reaching the most posterior portion of the vomer. The medial ridge is produced by the extremely concave posterolateral portions of both pterygoids. CP (ch 74).
75. Pterygoids, extending laterally almost reaching the mandibular condyle facet: 0 = absent; 1 = present, the pterygoid contacts the medial edge of the mandibular condyle when is seem in ventral view; 2 = present, the pterygoids extends not only laterally to reach the outline of the mandibular condyle facet, but also posteriorly far from the level of the condyles. CP (ch 75). Ordered.
76. Supraoccipitals, crista supraoccipitalis: 0 = poorly developed; 1 = protruding significantly posterior to the foramen magnum. CP (ch 76).

77. Supraoccipitals, large supraoccipital exposure on dorsal skull roof: 0 = absent; 1 = present. CP (ch 77).
78. Supraoccipitals, horizontal crest in the crista supraoccipitalis: 0 = absent or poorly developed anteriorly; 1 = present, along the entire crista supraoccipitalis. CP (ch 78).
79. Exoccipitals, medial contact of exoccipitals dorsal to foramen magnum: 0 = absent; 1 = present. CP (ch 79).
80. Basioccipital, morphology of the anteriormost part of the basioccipital: 0 = with two or one ventral tubercle; 1 = tubercle absent. CP (ch 80).
81. Basioccipital, deep C-shaped concavity between basioccipital tubera: 0 = absent; 1 = present. CP (ch 81).
82. Prootic, dorsal exposure: 0 = large; 1 = very reduce or absent. CP (ch 82).
83. Opisthotics, wide transverse occipital plane with depression for the nuchal musculature: 0 = absent; 1 = present. CP (ch 83).
84. Opisthotics, ventral ridge on opisthotic: 0 = absent; 1 = present, with an incipient enclosed middle ear region; 2 = present, but modified with an enclosed middle ear region. CP (ch 84).
85. Opisthotics, procesus interfenestralis: 0 = present, but not reaching the floor of cavum acustico-jugulare; 1 = present, reaching the floor of the cavum acusticojugulare but small; 2 = present, reaching the floor of the cavum acustico-jugulare but robust. CP (ch 85) Ordered.
86. Basisphenoid, rostrum basisphenoidale: 0 = flat; 1 = rod-like, thick and rounded. CP (ch 86).
87. Basisphenoid, paired pits on ventral surface of basisphenoid: 0 = absent; 1 = present. CP (ch 87).
88. Basiphenoid, ventral surface: 0= flat to slightly convex, with posterior margin straight or slightly concave; 1= V-shaped crest, with posterior margin forming the basipterygoid process projected posterolaterally. CP (ch 88).
89. Basiphenoid, rough surface between basisphenoid and basioccipital: 0 = absent; 1 = present. CP (ch 89).
90. Basiphenoid, dorsum sellae: 0 = low; 1 = high. CP (ch 90).
91. Basisphenoid, foramen anterius canalis carotici palatinum larger than foramen anterius canalis carotici cerebralis: 0 = absent; 1 = present. Modified from CP (ch 91).
92. Basiphenoid, foramen anterius canalis carotici cerebralis visible in dorsal anterior view of basisphenoid: 0 = widely separated; 1 = close together. CP (ch 92).
93. Hyomandibular, path of hyomandibular branch of the facial nerve: 0 = hyomandibular nerve passes through cranioquadrate space parallel to vena capitis lateralis; 1 = hyomandibular nerve runs independent from vena capitis lateralis. CP (ch 93).
94. Stapedial Artery, size of foramen stapedio-temporale: 0 = relatively large (≥5 mm diameter); 1 = significantly reduced in size (≤3 mm diameter); 2 = absent. CP (ch 94). Ordered.
95. Stapedial Artery, foramen stapedio-temporale location in the otic chamber: 0 = on dorsal part and pointing dorsally; 1 = on the anterior wall of the otic region, pointing anteriorly. CP (ch 95).
96. Recessus scalae tympani: 0 = almost nonexistent, not surrounded by bone; 1 = well developed. CP (ch 96).
97. Foramen jugulare posterius, relationship with the fenestra postotica: 0 = separate from fenestra postotica; 1 = coalescent with fenestra postotica. CP (ch 97).
98. Foramen nervi hypoglossi (XII), ventral covering: 0 = exposed in ventral view; 1 = covered in ventral view by an extension of the pterygoid and the basioccipital; 2 = covered in ventral view by an extension of the basioccipital. CP (ch 98).

99. Internal Carotid Artery, splitting of the internal carotid artery and the cerebral and palatine arteries: 0 = not embedded in braincase bone elements, the cerebral artery enters at the foramen posterius canalis carotici cerebralis (known previously as the foramen caroticum basisphenoidale) in the basisphenoid; 1 = partially embedded, the internal carotid artery enters in the braincase elements through the foramen posterius canalis carotici interni, running along the pterygoid canal, and then splitting into the cerebral and palatine arteries at the fenestra caroticus; 2 = fully embedded, the internal carotid artery enters in the braincase elements through the foramen posterius canalis carotici interni, and split inside the braincase, lack of a ventral exposed fenestra caroticus. CP (ch 99). Ordered.
100. Internal Carotid Artery, foramen posterius canalis carotici interni: 0 = absent; 1 = formed by pterygoid; 2 = formed by pterygoid and basisphenoid halfway along the basisphenoid-pterygoid suture; 3 = formed by prootic, prootic and basisphenoid, or prootic and pterygoid; 4 = formed by basisphenoid only. CP (ch 100).
101. Palatine Artery, entering in the skull: 0 = through the interpterygoid vacuity or intrapterygoid slit; 1 = through the foramen posterius canalis carotici palatinum between basisphenoid and pterygoid. Modified from CP (ch 101).
102. Fenestra Perilymphatica: 0 = large; 1 = reduced in size to that of a small foramen. CP (ch 102)
103. Cranial scutes, scute D meeting in midline: 0 = absent; 1 = present. CP (ch 103).
104. Cranial scutes, scute X much smaller than scute D: 0 = absent; 1 = present. CP (ch 104).
105. Cranial scutes, scute X partially separates scutes G: 0 = absent; 1 = present. CP (ch 105).
106. Cranial scutes, scutes A, B, and C forming a continus posterolateral shelf: 0 = absent; 1 = present. CP (ch 106).
107. Cranial scutes, scute F: 0 = formed by several scutes; 1 = formed by a single scute. CP (ch 107).
108. Cranial scutes, scute J: 0 = formed by several scutes; 1 = formed by a single scute. CP (ch 108).

Lower Jaw

109. Dentary, medial contact of dentaries: 0 = fused; 1 = open suture. CP (ch 109).
110. Dentary, width of triturating surface vs jaw length: 0 = narrow triturating surface, symphysis less than 1/3 of jaw length; 1 = broad triturating surface, symphysis ≥1/3 jaw length. CP (ch 110).
111. Dentary, symphyseal ridge: 0 = absent, flat triturating surface; 1 = present, but not visible in lateral view, flat to slightly convex triturating surface; 2 = present and greatly developed, visible in lateral view, ridge along entire length of symphysis. CP (ch 111). Ordered.
112. Dentary, lingual (tomial) ridge: 0 = prominent; 1 = weak or absent. CP (ch 112). Remarks: Cadena & Parham (2015) code *Ctenochelys stenoporus* as having a weak or absent tomial ridge of the dentary (1), but *Ctenochelys spp.* possesses a distinct tomial ridge, coded here as (0).
113. Dentary-Surangular arrangement: 0 = lack of a posterior expansion of dentary and anterior projection of surangular; 1 = posterior expansion of dentary present almost reaching the articular surface, covering the dorsal half of the surangular in lateral view, surangular with anterior projection. CP (ch 113).
114. Splenial: 0 = present; 1 = absent. CP (ch 114).

Carapace

115. Carapace, carapacial scutes: 0 = present; 1 = reduced not fully covering the carapace; 2 = absent. CP (ch 115). Ordered.

116. Carapace, three parallel lines of keels: 0 = absent; 1 = present, but only poorly developed; 2 = present and pronounced; 3 = present, but only with a medial line of keels on neurals, absence of keels on costals. CP (ch 116).

117. Shell, sculpturing of dorsal surface (carapace) and ventral surface (plastron): 0 = absent, smooth to slightly rugose; 1 = present, development of striations, vermiculations, striations, or pitting. CP (ch 117).

118. Shell, pattern of sculpturing of the dorsal surface (carapace) and ventral surface (plastron): 0 = parallel to radial striations; 1 = vermiculation; 2 = highly dense pattern of pitting combined with striations; 3 = dichotomic striations; 4 = spread pitting without marked striation pattern; 5 = granules (positive relief). CP (ch 118).

119. Carapacial Sutures: 0 = carapacial elements finely sutured or the contact is smooth; 1 = carapacial sutures strongly serrated in adult stage. CP (ch 119).

120. Nuchal, articulation of nuchal with neural spine of eighth cervical vertebra: 0 = cervical articulates with nuchal along a blunt facet; 1 = articulation absent; 2 = cervical articulates with nuchal along a raised pedestal. CP (ch 120).

121. Nuchal, elongate costiform process: 0 = absent; 1 = present, crosses peripheral 1; 2 = present, well developed reaches peripherals 2 or 3. CP (ch 121). Ordered.

122. Nuchal, length versus width: 0 = wider than long; 1 = longer than wide or as long as wide. CP (ch 122).

123. Nuchal, posteromedial fontanelles: 0 = absent; 1 = present. CP (ch 123).

124. Neurals, neural formula 6>4<6<6<6<6: 0 = absent; 1 = present. CP (ch 124).

125. Neurals, shape of neurals: 0 = very irregular in shape, wider than long or squared; 1 = regular, often perfectly hexagonal or pentagonal, often longer than wide. CP (ch 125).

126. Neurals, number of neurals: 0 = ten or more; 1 = nine or less; 3 = all neurals lost even in ventral view. CP (ch 126).

127. Peripheral Gutter: 0 = peripheral gutter absent or only anteriorly developed; 1 = peripheral gutter extensively developed along anterior and bridge peripherals. CP (ch 127).

128. Peripherals, number of peripherals: 0 = more than 11 pairs of peripherals present; 1 = 11 pairs of peripherals present; 2 = 10 pairs of peripherals present; 3 = less than 10 pairs of peripherals present. CP (ch 128). Ordered.

129. Peripherals, anterior peripherals incised by musk ducts: 0 = absent; 1 = present. CP (ch 129).

130. Costals, medial contact of the first pair of costals: 0 = absent; 1 = present. CP (ch 130).

131. Costals, medial contact of posterior costals: 0 = absent; 1 = medial contact of up to three posterior costals present; 2 = medial contact of all costals present. CP (ch 131). Ordered.

132. Costals, distal rib end and lateral ossification of the costal: 0 = costals fully ossified laterally with strong sutural contact with peripherals, lack of dorsal exposure of distal end of costal ribs; 1 = costals fully ossified laterally with strong sutural contact with peripherals, distal end of costal ribs exposed on dorsal surface and surrounded by the peripheral; 2 = costals lack lateral ossification, allowing the dorsal exposure of the distal end of ribs and the development of fontanelles only at the most anterior and posterior costals; 3 = costals with extreme loss of lateral

ossification, allowing the dorsal exposure of the distal end of ribs, in almost all series of costals. CP (ch 132).
133. Rib free peripherals: 0 = present, only anterior and posterior to ribs; 1 = present, between sixth and seventh ribs; 2 = present, between seventh and eighth ribs. CP (ch 133).
134. Costals, alternative short and long ends in the lateral part of costals: 0 = absent; 1 = present. CP (ch 134).
135. Costals, costal 9: 0 = present; 1 = absent. CP (ch 135).
136. Costals, shape of Costal 3: 0 = tapering towards the lateral side of the shell or with parallel anterior and posterior borders; 1 = broadens towards the lateral side of the shell. CP (ch 136).
137. Suprapygals, number of suprapygals: 0 = one; 1 = two; 2 = more than two; 3 = absent. CP (ch 137). Ordered.
138. Suprapygals, size between suprapygal 1 and 2: 0 = suprapygal 1 smaller than suprapygal 2; 1 = suprapygal 1 larger. CP (ch 138). Remarks: turtles with only one suprapygal or suprapygals absent are coded as (-).
139. Cervical scute: 0 = more than one cervical scute present; 1 = one cervical scute present; 2 = cervical scutes absent, carapacial scutes otherwise present. CP (ch 139).
140. Pygal, posterior notch: 0 = present; 1 = absent. CP (ch 140).
141. Supramarginals: 0 = complete row present, fully separating marginals from pleurals; 1 = partial row present, incompletely separating marginals from pleurals; 2 = absent. CP (ch 141). Ordered.
142. Vertebrals, shape of the verterbrals: 0 = vertebrals 2 to 4 significantly broader than pleurals; 1 = vertebrals 2 to 4 as narrow as, or narrower than, pleurals. CP (ch 142).
143. Vertebrals, position of vertebral 3-4 sulcus in taxa with five vertebrals: 0 = sulcus positioned on neural 6; 1 = sulcus positioned on neural 5. CP (ch 143).
144. Vertebrals, vertebral 3-4 sulcus with a wide posteriorly oriented medial embayment: 0 = absent; 1 = present. CP (ch 144).
145. Vertebrals, verebral 1: 0 = vertebral 1 does not enter anterior margin of carapace; 1 = enters anterior margin. CP (ch 145).
146. Marginals, marginal scutes overlap onto costals: 0 = absent, marginals restricted to peripherals; 1 = present. CP (ch 146).
147. Pleurals, at least one pair of additional pleural scutes located laterally of vertebral scute 1, with anterior contact with cervical scute: 0 = absent; 1 = present. CP (ch 147).

Plastron

148. Plastron, connection between carapace and plastron: 0 = osseous; 1 = ligamentous. CP (ch 148).
149. Plastron, central plastral fontanelle: 0 = absent; 1 = present. CP (ch 149).
150. Plastron, posterior plastral fontanelle, posterior plastral fontanelle between the xiphiplastra and/or the hypoplastra: 0 = absent in adult stage; 1 = retained in adult stage. CP (ch 150).
151. Plastron, plastral kinesis: 0 = absent, scutes sulci and bony sutures do not overlap; 1 = present, scutes sulci coincide with epiplastral-hyoplastral contact. CP (ch 151).
152. Plastron, plastral kinesis: 0 = between hyoplastron and hypoplastron; 1 = between hyoplastron and epiplastron/entoplastron. CP (ch 152).
153. Plastron, hyo-hypoplastra contact and shape: 0 = deep U or V-shaped axillar and inguinal notches, contact between hyo-hyoplastra absent or reduced due to the presence of mesoplastra or

a central fotanelle; 1 = deep axillar and inguinal notches, reduced contact between both elements due to the existence of central and lateral fontanelles; 2 = deep axillar and inguinal notches, extensive contact between hyo-hyoplastra (even for those taxa with plastral kinesis); 3 = a very narrow to absent contact between each other, star-shaped with extremely serrate medial edges, very shallow axillar and inguinal notches, and long lateral edges; 4 = extreme loss of ossification of hyo-hypoplastra, lack of contact between each other. CP (ch 153).
154. Entoplastron: 0 = present; 1 = absent. CP (ch 154).
155. Entoplastron, anterior entoplastral process: 0 = present, medial contact of epiplastra absent; 1 = absent, medial contact of epiplastra present. CP (ch 155).
156. Entoplastron, size of the posterior entoplastral process: 0 = posterior process long, reaching as far posteriorly as the mesoplastra; 1 = posterior process reduced in length. CP (ch 156).
157. Entoplastron, distinct posterolateral process: 0 = present; 1 = absent. CP (ch 157).
158. Entoplastron, shape of the entoplastron in ventral view: 0 = dagger-shaped; 1 = massive diamond-shaped; 2 = T-shaped, longer than wide; 3 = T-shaped, wider than long, forming broad lateral wings; 4 = strap like and V-shaped. CP (ch 158).
159. Entoplastron, suture with hyoplastra: 0 = tightly sutured; 1 = lightly sutured to almost absent contact between both. CP (ch 159).
160. Epiplastra, shape and contact of epiplastra: 0 = epiplastra squarish in shape, lack a contact between each other due to the narrow participation of the entoplastron in the anterior plastral lobe edge; 1 = epiplastra elongate in shape, with medial contact located anterior to the entoplastron; 2 = epiplastra squarish in shape lack of medial contact due to the extensive anterior and lateral projections of the entoplastron. CP (ch 160).
161. Epiplastra, very thick anterior lip in dorsal view: 0 = present; 1 = absent. CP (ch 161).
162. Hyoplastra, contacts of axillary buttresses: 0 = absent to slightly contacting peripherals only; 1 = peripherals and costal 1. CP (ch 162).
163. Hyoplastra, termination of axillary buttresses: 0 = terminates on peripheral 1 or 2; 1 = terminates on peripheral 3; 2 = terminates on peripheral 4 or 5 level; 3 = ossified axillary buttresses absent. CP (ch 163).
164. Mesoplastron: 0 = two present; 1 = one present; 2 = absent. CP (ch 164). Ordered.
165. Mesoplastron, medial contact of mesoplastra: 0 = present, or virtually present when a central plastral fontanelle is present, absence of contact between hyoplastron and hypoplastron; 1 = absent, partial contact between hyoplastron and hypoplastron present. CP (ch 165).
166. Hypoplastra, contacts of inguinal buttresses: 0 = absent to slightly contacting peripherals; 1 = peripheral and costal 5; 2 = peripheral, costals 5 and 6; 3 = peripherals and costal 4. CP (ch 166).
167. Hypoplastra, termination of inguinal buttresses: 0 = peripheral 8; 1 = peripheral 7; 2 = peripheral 6. CP (ch 167). Ordered.
168. Xiphiplastra, distinct anal notch: 0 = absent; 1 = present. CP (ch 168).
169. Xiphiplastra, shape of xiphiplastra: 0 = almost triangular to trapezoidal, with lateral straight to convex margin; 1 = rectangular elongated in shape, coupled forming together with the hypoplastron a very narrow posterior plastral lobe; 2 = narrow struts, separated by the posterior fontanelle. CP (ch 169).
170. Plastral scutes: 0 = present; 1 = absent. CP (ch 170).
171. Plastral scutes, midline sulcus: 0 = straight; 1 = distinctly sinuous, at least for part of its length. CP (ch 171).
172. Gular, number of gulars: 0 = one pair of scutes; 1 = only one scute. CP (ch 172).

173. Extragulars: 0 = present; 1 = absent. CP (ch 173).
174. Extagulars, medial contact: 0 = absent; 1 = present, contacting one another anterior to gular(s); 2 = present, contacting one another posterior to gular(s). CP (ch 174).
175. Extragulars, anterior plastral tuberosities: 0 = present; 1 = absent. CP (ch 175).
176. Extragulars, restricted to epiplastra: 0 = present; 1 = absent, extragulars reach the entoplastron. CP (ch 176).
177. Intergulars: 0 = absent; 1 = present. CP (ch 177).
178. Humerals, number of pairs: 0 = one pair present; 1 = two pairs present, subdivided by a plastral hinge. CP (ch 178).
179. Humerals, humero-pectoral sulcus: 0 = restricted to hyoplastra; 1 = crossing the posterior portion of entoplastron. CP (ch 179). Remarks: in extant cheloniids, this character is polymorphic, depending of the length of the posterior process of the entoplastron. This could also be the condition for most marine forms for which this character cannot be coded due to poor illustrations or bad preservation of sulci.
180. Pectorals: 0 = present; 1 = absent. CP (ch 180).
181. Pectorals, antero-posteriorly developed: 0 = present; 1 = absent, very short antero-posterior development. CP (ch 181).
182. Abdominals: 0 = present, in medial contact with one another; 1 = present, medial contact absent; 2 = absent. CP (ch 182). Ordered.
183. Anals: 0 = only cover parts of the xiphiplastra; 1 = overlap anteromedially onto the hypoplastra. CP (ch 183).
184. Inframarginals: 0 = more than two pair present, plastral scales do not contact marginals; 1 = two pair present (axillaries and inguinals), limited contact between plastral scales and marginals present; 2 = absent, unrestricted contact between plastral scales and marginal present. CP (ch 184). Ordered.

Neck

185. Cervical ribs: 0 = large cervical ribs present; 1 = cervical ribs reduced or absent. CP (ch 185).
186. Cervicals, position of the transverse processes: 0 = middle of the centrum; 1 = anterior end of the centrum. CP (ch 186).
187. Cervicals, posterior cervicals with strongly developed ventral keels: 0 = absent or slightly developed in all vertebrae; 1 = present, more developed on posterior vertebrae. CP (ch 187).
188. Cervicals, cervical 8 centrum significantly shorter than cervical 7: 0 = absent; 1 = present. CP (ch 188).
189. Cervicals, triangular diapophyses: 0 = absent; 1 = present. CP (ch 189).
190. Cervicals, central articulations of cervical vertebrae: 0 = articulations not formed, cervical vertebrae amphicoelous or platycoelous; 1 = articulations formed, cervical vertebrae procoelous or opisthocoelous. CP (ch 190).
191. Cervicals, articulation between cervical 8 and dorsal vertebrae 1: 0 = 8(dorsal 1; 1 = 8) dorsal 1; 1 = none, vertebrae only meet at zygapophyses. CP (ch 191).
192. Cervicals, biconvex cervical vertebrae in the middle of the neck: 0 = absent; 1 = present. CP (ch 192).
193. Cervicals, biconvex cervical vertebra in the middle of the neck: 0 = cervical 2; 1 = cervical 3; 2 = cervical 4; 3 = cervical 5. CP (ch 193).

194. Cervicals, biconcave cervical vertebrae: 0 = absent; 1 = present. CP (ch 194).
195. Cervicals, double articulation between cervical 5 and 6: 0 = absent; 1 = present. CP (ch 195).
196. Cervicals, double articulation between cervical 6 and 7: 0 = absent; 1 = present. CP (ch 196).
197. Cervicals, central articulation between cervical 6 and 7: 0 = cervical 6 concave (cervical 7 convex; 1 = platycoelous, cervical 6 II cervical 7. CP (ch 197).
198. Cervicals, double articulation between cervical 7 and 8: 0 = absent; 1 = present. CP (ch 198).
199. Cervicals, height versus length of centra and neural arch: 0 = total height of centra and neural arch longer than the anteroposterior length of the cervical centra; 1 = total height of centra and neural arch much shorter than the anteroposterior length of the cervical centra. CP (ch 199).
200. Cervicals, modification of neural arch on cervical 8: 0 = neural arch without modificiation of postzygapophyses; 1 = neural arch with postzygapophyses pointing anteroventrally. CP (ch 200).
201. Cervicals, postzygapophyses united in midline: 0 = absent; 1 = present. CP (ch 201).
202. Cervicals, ventral process on cervical 8: 0 = absent; 1 = present, well developed (as tall or taller than the height of the centrum). CP (ch 202).
203. Cervicals, shape of central articulation of cervicals 7 and 8: 0 = as high as wide; 1 = much wider than high. CP (ch 203).

Ribs

204. Ribs, length of first dorsal rib: 0 = long, extends full length of first costal and may even contact peripherals distally; 1 = intermediate, in contact with well-developed anterior bridge buttresses; 2 = intermediate to short, extends less than halfway across first costal. CP (ch 204). Ordered.
205. Ribs, contact of dorsal ribs 9 and 10 with costals: 0 = present; 1 = absent. CP (ch 205).
206. Dorsal rib 10: 0 = long, spanning full length of costals and contacting peripherals distally; 1 = short, not spanning farther distally than pelvis. CP (ch 206).

Vertebrae

207. Dorsals, anterior articulation of the first dorsal centrum: 0 = faces at most slightly anteroventrally; 1 = faces strongly anteroventrally. CP (ch 207).

Tail

208. Caudals, tail club: 0 = present; 1 = absent. CP (ch 208).
209. Caudals, caudal centra: 0 = all centra amphicoelous; 1 = all centra more or less pronounced procoelous; 2 = all centra more or less pronounced opisthocoelous; 3 = anterior few centra procoelous, posterior centra predominantly opisthocoelous. CP (ch 209).
210. Caudals, anterior caudal centra: 0 = amphicoelous; 1 = procoelous or platycoelous; 2 = opisthocoelous. CP (ch 210).
211. Caudals, posterior caudal centra: 0 = amphicoelous; 1 = procoelous or platycoelous: 2 = opisthocoelous. CP (ch 211).

212. Caudals, chevrons: 0 = present on nearly all caudal vertebrae; 1 = absent, or only poorly developed, along the posterior caudal vertebrae. CP (ch 212).
213. Caudals, tail ring: 0 = absent; 1 = present. CP (ch 213).

Pectoral girdle

214. Scapula, anterodorsal ridge of acromion: 0 = present; 1 = absent. CP (ch 214).
215. Scapula, ventral ridge of acromion: 0 = present; 1 = absent or only developed proximally near glenoid. Reworded from CP (ch 215).
216. Scapula, horizontal ridge of acromion: 0 = well-developed, coracoid foramen present; 1 = reduced, only developed along distal portion of acromion. CP (ch 216). Ordered.
217. Scapula, glenoid neck on scapula: 0 = absent; 1 = present. CP (ch 217).
218. Scapula, lamina between the dorsal process of the scapula and the acromion: 0 = well developed; 1 = reduced; 2 = absent. CP (ch 218). Ordered.
219. Scapula, internal angle between acromion process and scapular process ≥110o: 0 = absent; 1 = present. CP (ch 219).
220. Coracoid, coracoid vs humerus length: 0 = shorter than humerus; 1 = at least as long as humerus. CP (ch 220).
221. Coracoid, foramen: 0 = present; 1 = absent. CP (ch 221).
222. Cleithrum: 0 = present and in contact with the carapace; 1 = present, osseous contact with carapace absent; 2 = absent. CP (ch 222). Ordered.

Pelvic girdle

223. Pelvis, pelvis-shell attachment: 0 = pelvis-shell attachment by ligaments; 1 = pelvis attached by strong sutural contact of the ischium and pubis with the plastron, and illium with the carapace. CP (ch 223).
224. Pelvis, thyroid fenestra: 0 = coalescent; 1 = two separated fenestra completely or partially separated. CP (ch 224).
225. Ilium, elongated iliac neck: 0 = absent; 1 = present. CP (ch 225)
226. Ilium, iliac scar: 0 = extends from costals onto the peripherals and pygal; 1 = positioned on costals only. CP (ch 226).
227. Ilium, shape of the ilium articular site on the visceral surface of the carapace: 0 = narrow and pointed posteriorly; 1 = oval. CP (ch 227).
228. Ilium, posterior notch in acetabulum: 0 = absent; 1 = present. CP (ch 228).
229. Ilium, thelial process: 0 = absent; 1 = present. CP (ch 229).
230. Pubis, lateral process: 0 = small, poorly developed, columnar; 1 = well developed and flat. CP (ch 230).
231. Pubis, epipubis process: 0 = osseus or calcified; 1 = cartilaginous or absent. CP (ch 231).
232. Ischium, ischial contacts with plastron: 0 = contact via a large central tubercle; 1 = contact via two separate ischial processes. CP (ch 232).
233. Ischium, lateral process of ischium or metischial process: 0 = absent; 1 = present. CP (ch 233).
234. Hypoischium: 0 = present; 1 = absent. CP (ch 234).

Forelimb

235. Humerus, ectepicondylar foramen: 0 = in a channel; 1 = only a groove. CP (ch 235).
236. Humerus, proximal articular surface of humerus: 0 = with shoulder on preaxial side, upturned; 1 = without shoulder, not upturned. CP (ch 236).
237. Humerus, lateral process of humerus: 0 = abuts caput humeri; 1 = slightly separated from caput humeri; 2 = located distal to caput humeri but along proximal end of shaft; 3 = located at middle of humeral shaft. CP (ch 237). Ordered.
238. Humerus, lateral process of humerus: 0 = visible in dorsal view: 1 = not visible in dorsal view. CP (ch 238).
239. Humerus, lateral process shape: 0 = rounded to slightly squared; 1 = V-shaped or triangular. CP (ch 239).
240. Humerus, expansion of lateral process: 0 = limited to anterior surface of shaft; 1 = expands onto ventral surface. CP (ch 240).
241. Humerus, medial concavity of lateral process: 0 = absent; 1 = present. CP (ch 241).
242. Humerus, prominent anterior projection of lateral process: 0 = absent; 1 = present. CP (ch 242).
243. Humerus, length of the humerus versus the width of the proximal end: 0 = two times or less the width of the proximal end: 1 = more than two times the width of the proximal end. CP (ch 243).
244. Humerus, scar for Muscle latissimus dorsi and Muscle teres major: 0 = located anterior to humeral shaft; 1 = located at middle of shaft. CP (ch 244).
245. Humerus, humerus length vs femur length: 0 = shorter than femur; 1 = longer than femur. CP (ch 245).
246. Ulna, contact with radius through rugosity and ridge: 0 = absent; 1 = present. CP (ch 246).
247. Radius, curves towards anterior: 0 = absent; 1 = present. CP (ch 247).
248. Manus, phalangeal formula of the manus: 0 = most digits with two shortenened phalanges: 1 = most digits with three elongated phalanges. CP (ch 248).
249. Manus, paddles: 0 = absent, moveable articulations of all digit; 1 = 'half-paddle' digits 3 and 5 modified into paddle with rigid articulations. But digits 1 and 2 immoveable; 2 = elongate paddles present, digits 1 and 2 modified into paddle with rigid articulations, and very flat carpal and tarsal elements. CP (ch 249). Ordered.
250. Manus, flippers: 0 = absent; 1 = short flippers present; 2 = elongate flippers present. CP (ch 250). Ordered.
251. Ulnare, size of the ulnare vs the intermedium: 0 = smaller than intermedium: 1 = nearly as large as intermedium; 2 = much larger than intermedium. CP (ch 251). Ordered.

Hindlimb

252. Pes, number of digits: 0 = five; 1 = four. CP (ch 252).
253. Manus and Pes, flattening of carpals and tarsal elements: 0 = absent; 1 = present. CP (ch 253).
254. Manus and Pes, hyperphalangy manus digits 4 and 5, pes digit 4: 0 = absent; 1 = present. CP (ch 254).
255. Femur, femoral trochanters: 0 = distinct, and separated from one another; 1 = fossa obliterated, space between trochanters not concave, but notch present; 2 = fossa obliterated, trochanters connected by bony ridge without a notch. CP (ch 255). Ordered.

256. Tibia, tibial pit for pubotibialis and flexor tibialis internus muscles: 0 = absent; 1 = present. CP (ch 256).
