# Supplementary material for: A new species of Peritresius Leidy, 1856 (Testudines: Pan-Cheloniidae) from the Late Cretaceous (Campanian) of Alabama, USA, and the occurrence of the genus within the Mississippi Embayment of North America
Source: PLoS One. 2018 Apr 18;13(4):e0195651. doi: 10.1371/journal.pone.0195651 (PMC5906092; doi:10.1371/journal.pone.0195651)
Supplement: S4 File — Including (I) Locality age estimates; (II) Literature cited. (PDF) [file pone.0195651.s004.pdf]

(I) Age estimates for Late Cretaceous chelonioid *s.s.* localities appearing in Fig 11.

(1) DeGrey and Sharon Springs Members, Pierre Shale Formation of South Dakota: Both the DeGrey and Sharon Springs Members of the Pierre Shale of South Dakota have been dated to the middle-late Campanian. The DeGrey Member is interpreted as being upper Campanian based on a radiometric date of 74.58 Ma obtained from the underlying Crow Creek Member (Hanczaryk 2004) while the Sharon Springs Member has been dated to 80 Ma or middle Campanian (Obradovich 1993). **(2) Niobrara Formation of Kansas:** Based on ammonite and inoceramid bivalve zonation, the Niobrara Formation of Kansas ranges from uppermost Turonian (88.8 Ma) to lower Campanian (80.6 Ma) in age (Kauffman *et al.*, 1993). **(3) Marlbrook Marl Formation of Arkansas:** Planktonic foramineferal assemblages and ostracode zonation both indicate an upper Campanian age (76 to 72 Ma) for the Marlbrook Marl of southwestern Arkansas (Pessagno 1969). **(4) Coon Creek Member, Ripley Formation of Mississippi:** The presence of early Maastrichtian ammonites in the Coon Creek Member of Mississippi suggests a lower Maastrichtian (72 to 70 Ma) age for this portion of the Ripley Formation (Cobban and Kennedy 1993). **(5) Mooreville Chalk, Demopolis Chalk, and Ripley Formations of Alabama:** The time-transgressive Mooreville Chalk of central and western Alabama is considered upper Santonian (86 Ma) to middle Campanian (79.5 Ma) based on planktonic foramineferal biostratigraphy, as well as ostracode and nannoplankton zonation (Mancini *et al.* 1996; Mancini and Puckett 2005; Puckett 2005). The same methods were used to establish a range of 79.5 to 76 Ma or middle to upper Campanian for the Demopolis Chalk and upper Campanian (73.5 Ma) to lowest Maastrichtian (~71 Ma) for the Ripley Formation of Alabama

(Mancini and Puckett 2005; Puckett 2005). **(6) Severn Formation of Maryland:** Nannofossil zonation along with ostracode, gastropod, and ammonite assemblages indicate an upper Maastrichtian age (69 to 66 Ma) for the Severn Formation of Maryland **(**Kennedy *et al*. 1997). **(7) Navesink and Hornerstown Formations of New Jersey:** Though there appears to be a considerable amount of uncertainty surrounding the precise ages of both the Navesink and Hornerstown, vertebrate assemblages along with nanofossil zonation seem to indicate an upper Maastrichtian (68 Ma) to lower Paleogene (63.5 Ma) age for these formations (Miller *et al.*, 2004; Obasi *et al.* 2011).

(II) Literature cited

1. Hanczaryk P. Stratigraphy and paleoecology of the Middle Pierre Shale along the Missouri River (Central South Dakota). Geological Society of America, Rocky Mountain Section, Abstracts with Programs. 2004; 36: 67-68.
2. Obradovich J. A Cretaceous time scale. In: Caldwell W and Kauffman E. editors. Evolution of the Western Interior Basin. Geological Association of Canada, Special Paper 39. 1993;
3. Kauffman E, Sageman B, Kirkland J, Elder W, Harries P, Villamil T. 1993. Molluscan biostratigraphy of the Cretaceous Western Interior Basin, North America. In: Caldwell, W and Kauffman E. editors. Evolution of the Western Interior Basin, Geological Association of Canada, Special Paper 39. 1993; 397-434.
4. Pessagno E. Upper Cretaceous stratigraphy of the Western Gulf Coast area of Mexico, Texas, and Arkansas. Geological Society of America Memoirs. 1969; 111: 139 pp.

5. Mancini E and Puckett T. Jurassic and Cretaceous Transgressive-Regressive (T-R) Cycles, Northern Gulf of Mexico, USA. Stratigraphy. 2005; 2: 31-48.
6. Kennedy W, Cobban W, Landman N. Maastrichtian ammonites from the Severn Formation of Maryland. American Museum Novitates. 1997; 3210: 30 pp.
7. Miller K, Sugarman P, Browning J, Kominz M, Olsson R, Feigenson M, Hernandez J. Upper Cretaceous Sequences and Sea-Level History, New Jersey Coastal Plain. Bulletin of the Geological Society of America. 2004; 116: 368-393.
8. Obasi C, Terry D, Myer G, Grandstaff D. Glauconite Composition and Morphology, Shocked Quartz, and the Origin of the Cretaceous(?) Main Fossiliferous Layer (MFL) in Southern New Jersey, U.S.A. Journal of Sedimentary Research. 2011; 81: 479-494.
